# Integrating marker-assisted identification and multi-environment trait stability models to select superior rice restorer lines for hybrid breeding

**DOI:** 10.3389/fpls.2026.1781490

**Published:** 2026-03-11

**Authors:** Shridhar Ragi, Vikram Jeet Singh, Shekharappa Nandakumar, Subbaiyan Gopala Krishnan, Haritha Bollinedi, Ranjith Kumar Ellur, Kunnummal Kurungara Vinod, Bheemapura Shivakumar Harshitha, Sonu, Deepak Saran, Saurabh Samdarshi, Firos T. M. Basha, P. Jayaprakash, Deepak Singh Bisht, Ashok Kumar Singh, Prolay Kumar Bhowmick

**Affiliations:** 1 Division of Genetics, Indian Council of Agricultural Research (ICAR)-Indian Agricultural Research Institute, New Delhi, India; 2 Department of Seed Science and Technology, Acharya Narendra Deva University of Agriculture & Technology, Ayodhya, India; 3 Department of Genetics and Plant Breeding, Indian Council of Agricultural Research (ICAR)-Central Arid Zone Research Institute Jodhpur, Regional Research Station, Jaisalmer, Rajasthan, India; 4 Central Silk Board Department of Genetics and Plant Breeding, (CSB)-Institute for Seri-Biotechnological Research, Bangalore, India; 5 Department of Genetics and Plant Breeding, New Mexico State University, ASC at Clovis, NM, United States; 6 Division of Genetics, Indian Council of Agricultural Research (ICAR)-Rice Breeding and Genetics Research Centre, Aduthurai, Tamil Nadu, India; 7 Department of Plant Biotechnology, Indian Council of Agricultural Research (ICAR)-National Institute for Plant Biotechnology, New Delhi, India

**Keywords:** hybrid rice, multi-environment, multi-trait selection models, rice restorers, stability, WAASBY

## Abstract

The efficient identification of stable and agronomically superior restorer lines is critical for hybrid rice breeding, particularly under multi-environment testing where genotype × environment interaction confounds the selection decision. The present study aimed to integrate marker-assisted fertility restoration screening for *Rf3* and *Rf4* and multi-environment-, multi-trait-based selection models to identify elite rice restorer lines suitable for hybrid breeding. A panel of 240 rice restorer lines was screened using molecular markers for *Rf3* and *Rf4*, which revealed that 85.41% lines carried the *Rf4* allele, 11.25% lines possessed both *Rf3* and *Rf4*, and 3.34% lines carried only the *Rf3* allele. Then, these restorers are evaluated in multi-location for grain yield and associated agronomic traits. The WAASBY-based ranking identified superior genotypes combining high yield and stability, and genotypes selected under a 10% selection intensity were also present in quadrant IV of the Y × WAASB biplot, indicating above-average yield and high stability. Coincidence analysis across all stability and multi-trait selection models identified eight restorer lines (RR130, RR121, RR72, RR140, RR196, RR79, RR23, and RR233) as common restorers. These restorers showed mid-early flowering duration, high spikelet fertility, favorable panicle exertion, and superior seed yield per plant. The integrated selection strategy adopted in this study provides a practical basis for identifying elite restorer (R-line) parents for the development of high-yielding and widely adapted hybrid rice cultivars. To our knowledge, no prior research has concurrently utilized MGIDI, MTSI, MTMPS, and FAI BLUP for the identification of rice restorer lines in multi-environment trials.

## 1 Introduction

Rice (*Oryza sativa* L.) is the most widely consumed cereal crop and serves as the primary source of calories for more than half of the world’s population (Singh et al., 2024). In Asia, where nearly 90% of the global rice is produced and consumed, rising population pressure, shrinking arable land, and climate change continue to threaten sustainable food production (Singh et al., 2021; FAO, 2024; Nandakumar et al., 2025). India, the second-largest producer and consumer of rice, need to enhance rice productivity to meet future food security demands (Nandakumar et al., 2024). Hybrid rice technology remains one of the most effective strategies to break the yield plateau and enhance productivity by 15%–20% over conventional varieties (Virmani et al., 2003; Zeng et al., 2017). The majority of the hybrids produced in the world and all the hybrids in India are based on the cytoplasmic genetic male sterility (CGMS) system (Kumar et al., 2017a). However, the success of hybrid rice depends critically on the availability of genetically diverse, agronomically superior, and stable restorer lines capable of producing high-heterosis hybrids across diverse environments (Nematzadeh and Sattari, 2003).

Fertility restoration of the WA system is governed by fertility restoring (*Rf*) genes that are naturally available in the rice gene pool. As many as 17 Rf genes are reported in rice compatible for various CMS systems. For the WA system, two genes, *Rf_3_* mapped on the short arm of chromosome 1 (Zhang et al., 1997) and *Rf_4_* located on the long arm of chromosome 10 (Yao et al., 1997), are recognized as the most potential restoration genes (Alavi et al., 2009). In earlier days, phenotyping followed by hybridization was the only method used for identifying potential rice restorers, which was tedious, time‐consuming, and expensive and had limited outcome. Presently, molecular markers linked to *Rf* loci have been identified and are being used for high throughput screening of rice lines for potential restoration ability based on the presence of *Rf_3_* and/or *Rf_4_* loci (Shidenur et. al., 2019). Restorer lines developed from *indica* × tropical *japonica* hybridization, iso-cytoplasmic derivatives, landraces, and elite commercial cultivars offer genetic diversity for parental line selection to develop superior hybrids (Kallugudi et al., 2022: Singh et al., 2022). The iso-cytoplasmic restorer lines are unique as they carry the same male sterile cytoplasm which minimizes the potential cyto-nuclear conflict between the cytoplasmic and nuclear genes. Additionally, these iso-cytoplasmic restorers carry male sterile cytoplasm, and therefore there is no need for test-crossing to assess their restoration potential. The information on the status of restorer genes *Rf_3_* and *Rf_4_* in the iso-cytoplasmic restorers can help in estimating the efficiency of these genes, either alone or in combination, in restoring fertility in the test-cross hybrids in different environments (Yao et al., 1997; Kumar et al., 2017b). However, the performance of the hybrids is influenced by quantitative traits, which makes the selection of superior restorer lines challenging. Moreover, genotype × environment interaction (GEI) often complicates true genetic potential, necessitating analytical frameworks that can simultaneously evaluate multi-trait performance and stability across multiple environments. Assessing yield stability requires evaluations over multiple years and locations, as single-field experiments in 1 year are insufficient (Reckling et al., 2021). Direct selection for these traits may be ineffective due to their low heritability, which limits genetic progress.

Understanding genotype × environment interaction in multi-environment trials (METs) is essential for identifying genotypes that consistently perform well across varying conditions (Chandra et al., 2022). In analyzing MET data, the additive main effects and multiplicative interaction (AMMI) model and genotype and genotype-by-environment (GGE) biplots are commonly used for identifying genotypes that perform well across location (Sanjana Reddy et al., 2021; Asungre et al., 2022). However, both models fail to account for genotypic random effects and focus primarily on genotype–environment interactions, making them sensitive to environmental variability and affecting the consistency of genotype evaluations (Wodebo et al., 2023; Hossain et al., 2023; Khandelwal et al., 2024). Additionally, linear selection indices often suffer from collinearity among traits, which can lead to biased multiple regression coefficients and reduced selection effectiveness (Silva et al., 2021). Exploratory methods such as principal component analysis (PCA) and linear discriminant analysis (LDA) are useful for dimensionality reduction and visualizing trait relationships in multi-environment research (Ekka et al., 2021). Despite their benefits, these techniques face challenges in effectively ranking the genotypes based on trait values. To overcome this, a linear mixed model (LMM)- based quantitative stability index, weighted average of absolute scores from the singular value decomposition (SVD) of the matrix of best linear unbiased prediction [(BLUPs) WAASB], was developed to study GEI through biplots for the identification of stable genotypes (Olivoto et al., 2019a). The weighted average absolute scores from BLUP for yield (WAASBY), a superiority index, was introduced to combine the mean performance with the WAASB stability score for genotype selection (Olivoto et al., 2019a). Recent advances in quantitative genetics have emphasized the need for selection approaches that jointly account for multiple traits and genotype × environment interaction, particularly in hybrid rice breeding programs where restorer performance must be stable across locations. Multi-trait selection indices have therefore emerged as effective alternatives to single-trait or yield-focused methods, as they integrate trait interrelationships, stability, and breeding objectives within a unified framework (Olivoto and Nardino, 2021; Olivoto et al., 2019b; Rocha et al., 2018). Unlike conventional stability analyses, these indices align selection with the ideotype concept, enabling the identification of genotypes that simultaneously satisfy agronomic performance, stability, and target trait profiles. However, their application for the systematic identification of elite rice restorers across multiple environments remains limited. In this context, the present study evaluates the utility of complementary multi-trait and multi-environment selection indices to identify stable and agronomically superior restorer lines. The reliable and comprehensive selection of elite restorer lines contributes to the development of parental restorer lines for high-yielding and widely adapted hybrid cultivars.

## 2 Materials and methods

### 2.1 Plant material and multi−environment trials

A panel of 240 diverse rice restorer lines was used in the current study to identify potential rice restorers. The panel consisted of 198 iso-cytoplasmic restorer lines derived from popular rice hybrids grown in India through generation advancement, followed by selection for desirable agronomic traits and spikelet fertility (Kumar et al., 2017a). A total of 42 tropical *japonica/indica* (TRJ)-derived lines were developed by crossing Pusa 44 (a popular non-aromatic indica rice variety) with 29 diverse tropical *japonica* lines sourced from the International Rice Research Institute (IRRI) (Shidenur et al., 2019). All materials used in this study were developed at the Division of Genetics, ICAR-Indian Agricultural Research Institute (IARI), New Delhi, India. The 240 diverse restorers utilized in the current study were designated as RR, followed by a unique serial number (**Supplementary Table 1**).

The test panel was evaluated at three different locations, namely, the Division of Genetics, ICAR-IARI, New Delhi (DEL, 28°38′ N, 77°10′ E; 224 m), the IARI-Collaborative Outstation Research Centre (CORC), Rakhra, Punjab (RKR, 30°22′ N, 76°15′ E; 253 m) during kharif 2022, and at IARI-RBGRC, Aduthurai, Tamil Nadu (ADT, 11°00′ N, 79°28′ E; 23 m) during the rabi season of 2022–2023. Accordingly, the three test environments were coded as E1 (New Delhi, kharif 2022), E3 (Rakhra, kharif 2022), and E2 (Aduthurai, rabi 2022–23), representing contrasting rice-growing ecologies of India, including the north-central plains (representative of Haryana and western Uttar Pradesh), the north-western Indo-Gangetic plains, and the southern Cauvery delta region, respectively. The two locations, namely, Aduthurai and Delhi, were part of a shuttle breeding chain of a rice breeding program at ICAR-IARI. These locations are situated more than 2,500 km apart and known for diverse agroecology conditions (Nagarajan et al., 2013). Moreover, the evaluation was conducted across two distinct seasons, serving as a temporal control to identify restorer lines with broad adaptability to contrasting environmental conditions and weather parameters. Among three locations, the climatic condition of Rakhra and Delhi regions is similar to the hybrid rice geography of Punjab, part of Haryana, and western UP where hybrids are prevalent among farmers. The third location, Aduthurai, is known for less hybrid rice cultivation but selected as it offers a distinct environmental contrast to the northern sites. Sowing was undertaken on June 6, 2022 in E1, June 10, 2022 in E3, and December 20, 2022 in E2, with transplanting carried out 21 days after nursery sowing, respectively. Weather data (daily maximum and minimum temperature and rainfall) were obtained from the respective meteorological observatories at each location. The monthly means of maximum and minimum temperature and cumulative rainfall during the crop growing period are summarized in **Supplementary Table 7** to characterize the environmental conditions prevailing at each test environment.

The experiment was conducted using an alpha lattice design with two replications, each consisting of incomplete blocks with entries randomized within blocks. In E1 and E3, the soil types were sandy loam, whereas in E2, it was alluvial clay. At all sites, the experiment was conducted under irrigated transplanted conditions, and the nursery was raised on elevated beds for 21 days before the seedlings were transplanted into a well-puddled field. The crop was planted at a spacing of 15 × 15 cm between plants, with a 20-cm space maintained between rows in 1-m² plot, and was grown under fully irrigated conditions with the recommended dose of fertilizers (RDF) applied as per local recommendation across all environments. Weed management was carried out using recommended pre-emergence herbicides, and pest and disease incidences were managed following standard plant protection practices to avoid biotic stress. To minimize border effects, observations were recorded from plants in the central area of each plot, excluding border plants.

### 2.2 Morphological characterization

Data were recorded for various agro-morphological traits on five randomly selected plants for all restorer lines. Observations were recorded for days to 50% flowering (DFF, days) by counting the number of plants that flowered in each plot. Productive tiller number per plant (PTN) is measured by counting the number of tillers bearing panicles with filled grains in each plant. Plant height (PH) is taken by measuring the length of the plant from the ground level to the tip of the primary panicle. Panicle length (PL) is the length of primary panicles measured from the base of the peduncle to the tip of the panicle, excluding awns. The number of filled grains per panicle (FG) is taken by counting the number of filled grains in a primary panicle. Thousand seed weight (TSW) is measured by weighing 1,000 filled grains. Seed yield per plant (SYP) was taken by weighing the grains harvested from a plant, which were dried to an optimum moisture level of approximately 14%. Spikelet fertility (SF) is expressed as the percentage of the average ratio between the number of filled grains (FG), and the total number of grains per panicle was calculated from five representative plants (Raj and Virmani, 1988). The panicle exsertion ratio is expressed as a percentage. It is calculated using data on the length of the panicle from the flag leaf ligule to the tip of the panicle and the length of the panicle (PL). The ratio is determined using the following formula (Gangashetti et al., 2004; Harshitha et al., 2024):


$$\begin{array}{l} \text{Panicle exsertion ratio }(\text{PER})\\ =\frac{\text{length from flag leaf ligule to tip of the panicle}}{\text{Panicle length}}\times 100 \end{array}$$

### 2.3 Molecular characterization of restorer lines for fertility restorer genes (*Rf_3_* and *Rf_4_*)

Genomic DNA was isolated from the leaves of young seedlings of each plant using the standard CTAB procedure (Murray and Thompson, 1980). The extracted DNA was quantified using 0.8% agarose gel in 1× TAE buffer and the diluted, uncut genomic DNA as a standard. The test genotypes were screened for the presence or absence of the restorer alleles of *Rf_3_* and *Rf_4_* gene(s) using widely used SSR markers, namely, DRRM‐Rf3‐5 (*Rf_3_*) and RM6100 (*Rf_4_*) (Prakash, 2003; Suresh et al., 2012) (**Supplementary Table 2**). The polymerase chain reaction (PCR) for all the markers was carried out with steps of initial denaturation at 94 °C for 5 min, followed by 35 cycles of denaturation (at 94 °C for 30 s), annealing (at 55 °C for 30 s), and 72 °C for 1 min of extension. The final extension was carried out at 72 °C for 5 min. Finally, the PCR products were scored based on agarose gel electrophoresis. The PCR products were resolved on 3.5% metaphor^®^ agarose gel prepared by dissolving 17.5 g of fine agarose powder in 500 mL of 1X TAE buffer. The PCR products were run until the bands were clearly separated on the gel. The banding pattern was visualized using a gel documentation system (Bio-Rad Laboratories Inc., USA). To ensure genotyping accuracy, PCR amplification and allele scoring were repeated for samples showing ambiguous or weak banding patterns. Genotypes showing clear and reproducible amplification patterns were retained for classification. Heterozygous banding patterns were recorded separately and were not used for definitive restorer classification; such lines are intended for further selfing and re-screening in subsequent generations to fix homozygosity. The use of well-characterized restorer (PRR 78) and maintainer (Pusa 6B) checks across all PCR runs ensured the consistency and reliability of allele scoring.

### 2.4 Statistical analysis

#### 2.4.1 Variance component analysis

A pooled analysis of variance across environments was performed using the “anova_joint” function of the “metan” package in R (v.4.2.3) (Olivoto and Lúcio, 2020) to assess the significance of the random effects. A likelihood ratio test (LRT) was conducted using two-tailed chi-square test. The homogeneity of variance across the three environment was tested using Leven’s test (Levene, 1960), implemented in the “car” package (Fox and Weisberg, 2019). The mean trait performance of rice restorers across environments was depicted using boxplots constructed in R (v.4.2.3) with the “tidyverse**/**ggplot2” packages. Replication means were used for visualization, and environmental effects were assessed using one-way ANOVA followed by Tukey’s HSD test (*p* ≤ 0.05).

#### 2.4.2 Mixed-model analysis and BLUP estimation

Multi-environment morphological data were analyzed using linear mixed models fitted through the best linear unbiased prediction/restricted maximum likelihood (BLUP/REML), which is a standard approach for estimating variance components and enabling optimal selection in multi-environment trials (Olivoto et al., 2019a; Olivoto and Lúcio, 2020). In the current study, precision was enhanced by treating genotype, genotype–environment interaction, and incomplete blocks nested within complete replicates as random effects, allowing the utilization of inter-block information. Environments and complete replicates within environments were treated as fixed factors (Moehring et al., 2015; Olivoto et al., 2019a). Variance parameters were calculated using the standard linear mixed model described by Yang (2007).


y=Xβ+Zµ+∈

where *y* is a vector of the response variable (such as grain yield), *β* is a vector of fixed effects, *u* is a vector of random effects, *X* and *Z* are design matrices of 0 and 1 s relating *β* and *u* to *y*, respectively, and *ϵ* is a vector of random error.

The significance of random effects was tested using likelihood ratio tests (LRT). Variance homogeneity across environments was assessed using Levene’s test, and model adequacy was further evaluated through an inspection of residual Q–Q plots to verify normality and homoscedasticity, following standard mixed-model procedures implemented in the *metan* package. Best linear unbiased predictions (BLUPs) for genotypic main effects and GEI effects were extracted from the fitted models and used for subsequent stability and multi-trait selection analyses.

#### 2.4.3 Mean performance and stability of multiple traits

Genotype stability across the environments was quantified using the weighted average of absolute scores (WAASB) from the singular value decomposition (SVD) of the BLUP matrix for the GEI effects generated by a linear mixed model, as proposed by Olivoto et al. (2019a) for the individual genotypes.


$$WAASB_{i}\;=\;\frac{\sum_{k=1}^{P}|IPCA_{ik}-EP_{k}|\;}{\sum_{k=1}^{P}EP_{k}}$$

where WAASBi is the weighted average of absolute scores of the *i*-th genotype, IPCA_ik_ is the score of the *i*-th genotype in the *k*-th interaction principal component axis (IPCA), and EP_k_ is the amount of the variance explained by the *k*-th IPCA. The WAASB index summarizes the contribution of each genotype to GEI across all interaction component axes (IPCAs), with lower WAASB values indicating greater stability. To jointly consider mean performance and stability, the WAASBY index was computed by integrating rescaled mean performance and WAASB values, following Olivoto et al. (2019a).

Subsequently, both the BLUP-based mean performance of early maturity indicators and the WAASB index stability values were rescaled to the range of 0 to 100, where 0 depicts the most undesirable value (e.g., the genotype with the most days to first flowering will have the lowest rescaled value of 0) and 100 is for the most anticipated value (Olivoto et al., 2019a). The rescaled value of a given trait for the *i*-th genotype and the *j*-th trait for both mean performance (*rY_i_*) and stability (*rW_i_*) is given by Olivoto et al. (2019a). For traits such as DFF and PH, where negative gains are desired, we considered the original maximum and minimum values to be 0 and 100, respectively. However, for traits PER, TSW, FG, SF, PL, PTN, and SYP, where positive gains were desired, the original maximum and minimum values were considered to be 100 and 0, respectively. The genotype with a rescaling value of 100 for all the preferred traits represents the ideal genotype, following the concept proposed by Donald (1968).

### 2.5 Multi−trait−based stability evaluation methods

The rescaled BLUP means and stability values were subsequently used to compute multi-trait and multi-environment selection indices. The present study included four stability evaluation methods to check the efficiency of the methods and to identify the best restorer lines for further utilization in hybrid breeding. Data analysis was performed using R Studio with base R version 4.3.2. The study employed various functions in the “metan” package for comprehensive genotype assessment and stability evaluation, including gamem_met () for BLUP-based analyses, waasb () for WAASB scores, mgidi () for the MGIDI index, mtsi () for the MTSI index, FAI-BLUP () for the FAI-BLUP index, and mtmps () for the multi-trait mean performance and stability index. 

#### 2.5.1 Multi-trait genotype-ideotype distance index

The multi-trait genotype–ideotype distance index (MGIDI) was calculated following the methodology of Olivoto and Nardino (2021), which involves four steps: rescaling the traits, conducting factor analysis, designing the ideotype (which exhibits a rescaled value of 100 for all evaluated traits), and computing the MGIDI index.


$$MGIDI_{i}=\sqrt{\sum_{j=1}^{f}{\left(Y_{ij}\;-Y_{j}\;\right)}^{2}}$$

where MGIDI_i_ is the multi-trait genotype-ideotype distance index for the *i*-th genotype, γ^ij^ is the score of the *i*-th genotype in the *j*-th factor (*i* = 1, 2, *t*; *j* = 1, 2, *f*), with *t* and *f* being the number of genotypes and factors, and γ_j_ is the *j*-th score of ideotype.

The strength and the weakness of genotypes were assessed by calculating the proportion of the MGIDI of the *i*-th genotype explained by the *j*-th factor (ω_ij_) as follows (Olivoto et al., 2022).


$$\omega_{ij}=\frac{\sqrt{D_{ij}^{2}}}{\sum_{j=1}^{j}\sqrt{D_{ij}^{2}}}$$

where *D*_ij_ is the distance between the *i*-th genotype and the ideal genotype for the *j*-th factor.

#### 2.5.2 Multi-trait stability index

The multi-trait stability index (MTSI) differs from the MGIDI by incorporating WAASBY (mean performance and stability) values into the Fgp matrix through factor analysis, whereas the MGIDI incorporates only BLUP mean values (Behera et al., 2024). The new quantitative genotypic stability measure, WAASB, is the weighted average of absolute scores from the singular value decomposition of the matrix of BLUPs for the GEI effects generated by a linear mixed model.


$$WAASB_{i}\;=\;\frac{\sum_{k=1}^{P}|IPCA_{ik}-EP_{k}|}{\sum_{k=1}^{P}EP_{k}}$$

where WAASBi is the weighted average of absolute scores of the *i*-th genotype, IPCA_ik_ is the score of the *i*-th genotype in the *k*-th interaction principal component axis (IPCA), and EP_k_ is the amount of the variance explained by the *k*-th IPCA.

The WAASB index gives more weightage to stability only, whereas the WAASBY index employs both mean performance and stability (Nataraj et al., 2021; Olivoto et al., 2019a). By using WAASBY, the multi-trait stability index (MTSI) (Olivoto et al., 2019b) was calculated by using the following formula:


$$MTSI_{i}=\sqrt{\sum_{j=1}^{f}{\left(F_{ij}\;-F_{j}\;\right)}^{2}}$$

where the MTSI_i_ is the multi-trait stability index for the *i*-th genotype, *F_ij_* is the *j*-th score of the *i*-th genotype, and *F_j_* is the *j*-th score of the ideotype.

#### 2.5.3 Multi-trait mean performances and stability index

The multi-trait mean performances and stability index (MTMPS) is typically derived from the multi-trait stability index (MTSI) proposed by Olivoto et al. (2019b), which considers various parametric and non-parametric stability indices. In this study, MTMPS was calculated using Wricke’s ecovalence (W_i_) instead of the WAASBY index, marking the only difference from MTSI (Yue et al., 2022).

#### 2.5.4 Multi-trait index based on factor analysis and ideotype design

Once the ideotype is established, the spatial probability of each genotype is calculated by estimating its distance from the ideotype. This ranking assists in the evaluation of genotypes. The calculation formula for the factor analysis and ideotype design (FAI-BLUP) index is as follows (Rocha et al., 2018):


$$P_{ij}=[\frac{\left(\frac{1}{d_{ij}}\right)}{\sum_{i=1,j=1}^{i=n,j=m}(\frac{1}{d_{ij}})}]$$

where *P*_ij_ represents the probability that the *i*-th genotype (*i* = 1, 2,…, *n*) is similar to the *j*-th genotype (*j* = 1, 2,…, *m*), and *d*_ij_ represents the genotype–ideotype distance from the *i*-th genotype to the *j*-th ideotype according to the standardized average Euclidean distance.

#### 2.5.5 Selection differential (S) and genetic gain under selection (ΔG)

The selection differential, expressed as a percentage of the population mean, was computed for each trait as follows:


$$\;S\%=\frac{\left(X_{s}-X_{o}\right)}{X_{O}}\times 100$$

The percentage of genetic gain under selection (ΔG %) is calculated as follows:


$$\Delta G\%=\;\frac{\left(X_{s}-X_{o}\right)}{X_{O}}\times h\times 100$$

where *X*_o_ is the mean WAASBY index of the original population and *X*_s_ is the mean WAASBY index of the selected genotypes.

These models were employed to identify rice restorers that combine early maturity with stability across multiple traits. These indices are based on the ideotype concept, wherein traits are rescaled to a common scale (0–100) according to their desired direction, with 0 representing the least desirable value and 100 representing the most desirable value. This rescaling facilitates the definition of an ideotype, as originally proposed by Donald (1968), enabling the selection of genotypes closest to the ideal multi-trait profile.

#### 2.5.6 Definition of the target ideotype and trait desirability

The target ideotype was defined based on breeding objectives relevant to hybrid rice restorer development, emphasizing early to mid-early flowering (lower DFF), optimal plant height, and improved yield and yield-related traits, including SF, PER, PL, FG, TSW, and SYP. Trait desirability (increase or decrease) was specified *a priori* and is summarized in **Table 1**. All traits were rescaled to a common 0–100 scale according to their desired direction prior to index computation. No subjective weights were assigned to individual traits; factor analysis was instead used to group correlated traits, implicitly balancing trait contributions within each factor during index construction.

**Table 1:** Selection differential and selection gain for weighted average of absolute scores (WAASB) for each factor and variable among 240 rice restorers across the environments.

Trait	Factor	*X*_o_	*X*_s_	S	S%	ΔG	ΔG%	Indicator
PH	FA1	104	106	1.51	1.45	0.914	0.876	Decrease
PL	FA1	25.4	25.5	0.0922	0.363	0.0361	0.142	Increase
DFF	FA1	98.2	99.7	1.5	1.53	1.06	1.08	Decrease
PTN	FA2	11.4	11.7	0.246	2.16	0.0293	0.256	Increase
TSW	FA2	21.5	23	1.48	6.86	0.626	2.91	Increase
FG	FA2	161	158	-2.97	-1.84	-1.33	-0.824	Increase
SYP	FA3	22.3	22.7	0.334	1.49	0.00979	0.0438	Increase
SF	FA3	84.3	85.3	0.95	1.13	0.307	0.364	Increase
PER	FA4	96.4	97.1	0.654	0.678	0.0438	0.0454	Increase

*X*_o_, population mean; *X*_s_, mean of selected individuals; S, selection differential; S%, selection differential percentage; G, selection gain; ΔG%, selection gain percentage; DFF, days to 50% flowering (days); PH, plant height (cm); PER, panicle exertion ratio; PL, panicle length (cm); FG, filled grains per panicle; SF, spikelet fertility (%); PTN, productive tiller number; TSW, thousand seed weight (g); SYP, seed yield per plant (g).

## 3 Results

### 3.1 Molecular characterization and grouping of the restorer lines for *Rf3* and *Rf4* genes

**Figure 1:**
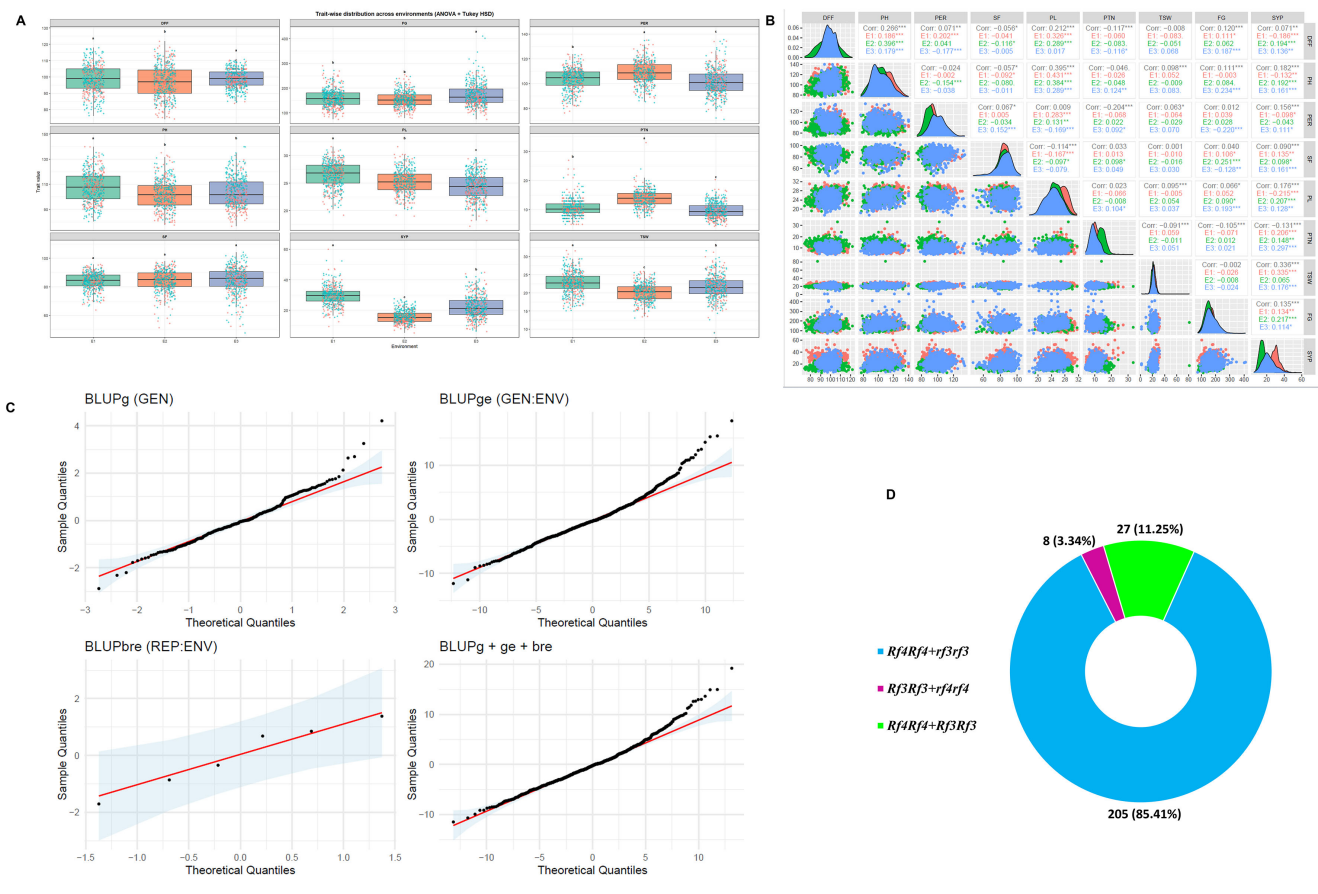
**(A)** Boxplots depicting the distribution of genotype mean performance for nine agronomic traits evaluated across three environments (E1, E2, and E3). The central line indicates the median, boxes represent the interquartile range, and whiskers denote data spread. Jittered points correspond to individual genotype observations. Environmental means sharing the same lowercase letter are not significantly different according to Tukey’s HSD test (*p* ≤ 0.05). **(B)** Correlations and frequency distributions of nine agronomic traits among 240 rice restorers across three different environments. DFF, days to 50% flowering (days); FG, filled grains; PER, panicle exertion ratio; PH, plant height (cm); PL, panicle length; PTN, productive tiller number; SF, spikelet fertility (%); TSW, thousand seed weight (gm); SYP, seed yield per plant (gm). **(C)** Quantile–quantile plots illustrating the distribution of block within replication (BLUPbre), genotype (BLUPg), total phenotypic effects (BLUPg+ge+bre), and genotype-by-environment interaction (BLUPge), all depicted with a 95% confidence interval for normality adjustment across various environments. **(D)** Frequency distribution of *Rf3* and *Rf4* genes in 240 rice restorers based on marker patterns.

A total of 240 rice restorer lines were screened for the presence of fertility restoration genes *Rf3* and *Rf4* using the widely employed SSR markers, DRRM-RF3–5 and RM6100, respectively. The allelic status of the restorer genes is given in the **Supplementary Table 3**. Among the 198 iso-cytoplasmic restorer lines (RR1 to RR198), seven lines contained both *Rf_3_* and *Rf_4_* genes, and the remaining 190 lines contained only the *Rf4* gene, while a single line carried only the *Rf_3_* gene. In tropical *japonica*-derived lines, out of 42 restorer lines (RR199–RR240), 20 lines carried both *Rf_3_* and *Rf_4_*, seven lines possessed only *Rf3*, and the remaining 15 lines contained only *Rf_4_*. Based on the amplification profiles, the restorers were classified into three categories, lines carrying only *Rf_3_*, only *Rf_4_*, and both *Rf_3_* and *Rf_4_*. Among the 240 restorers, 205 restorers (85.41%) had the functional *Rf_4_* allele, 27 (11.25%) lines had the both the *Rf_3_* and *Rf_4_* functional alleles, and additionally a small proportion—3.34% (eight lines)—of the restorer lines possessed only the *Rf_3_* allele (**Figure 1D**).

### 3.2 Mean performances and analysis variance

The pooled ANOVA indicated highly significant differences for restorer lines, with a significant effect of environments and G × E interaction (GEI) (*p* < 0.0001) for all of the studied traits, indicating substantial phenotypic variation among the 240 rice restorer lines across the three test environments for all of the traits studied. The least significant difference (LSD) values ranged from 3.68 for SYP, 30.31 for FG, and 8.71 for PH, respectively. Furthermore, the ratio of the mean squares of genotypes to error (MSR^+^/MSR^−^) was greater than unity for all traits, with values ranging from 1.22 for PH to 5.32 for SYP, indicating a predominance of positive genotype × environment interaction effects (**Table 2**). DFF ranged from 74 to 122 days, with a mean of 98.19 days, reflecting the availability of early to late maturing restorers. PH varied widely from 76.6 to 140.0 cm, while PL ranged from 17.33 to 31.60 cm among the restorers. The SF ranged from 47.76% to 94.33%, and FG varied widely from 74 to 410. TSW showed a wide range (9.04–31.70 g), whereas SYP varied from 5.16 to 60.0 g. The coefficients of variation (CV) were low to moderate for DFF (4.19%), PH (6.95%), PL (6.71%), PER (6.81%), and SF (6.1%). In contrast, higher CV values were recorded for PTN (16.65%), FG (9.92), TSW (9.46%), and SYP (13.84%) (**Table 3**). DFF showed a moderate distribution across environments, and FG exhibited a higher environmental influence, with significantly higher mean values observed in E3 compared to E1 and E2 (Tukey’s HSD, *p* ≤ 0.05). PH and PL also differed significantly across environments, with E1 generally supporting taller plants and longer panicles, whereas reduced values were recorded in E2. PTN was significantly higher in E2, and SF displayed relatively stable distributions across environments, although a wider range was evident in E3. SYP varied among environments, with the highest median values recorded in E1 (**Figure 1A**).

**Table 2:** Pooled analysis of variance with mean sum of squares for yield and yield-attributing traits in three environments.

Source	DFF	PH	PER	SF	PL	PTN	TSW	FG	SYP
ENV	400.3***	4292.2***	11046.41***	88.77*	536.9***	2145.69***	757.56***	42020***	22939.18***
REP(ENV)	68.2**	4212***	114.18***	8.49	72.5***	79.68***	3.84	38276***	560.84***
BLOCK(REP*ENV)	49.2***	123.7***	11.86	23.23	1.9	8.37***	6.86*	1565***	19.1**
GEN	239***	423.2***	105.23***	107.69***	12.2***	13.51***	28.09***	3246***	70.58***
GEN: ENV	44.6***	75***	156.92***	71.09***	3.7**	9.2***	12.4***	1684***	50.88***
Residuals	16.9	52.5	9.28	26.45	2.9	3.62	4.15	256	9.56
CV(%)	4.19	6.95	6.81	6.1	6.71	16.65	9.46	9.92	13.84
LSD	6.56	8.71	8.81	5.54	1.73	1.84	2.71	30.31	3.68
MSR+/MSR-	2.21	1.22	2.78	1.86	1.89	1.74	4.56	2.56	5.32

DFF, days to 50% flowering (days); PH, plant height (cm); PER, panicle exertion ratio; PL, panicle length (cm); SF, spikelet fertility (%); PTN, productive tiller number; TSW, thousand seed weight (g); FG, number of filled grains per panicle; SYP, seed yield per plant (g); CV (%), coefficient of variation in percentage; LSD, least significant difference; MSR+/MSR−, mean square ratio of positive vs. negative GEI effects.

***p < 0.001, ***p < 0.01, *p < 0.05.

**Table 3:** Mean performance, likelihood ratio test, genetic parameters, and variance components for nine morphological traits evaluated in 240 rice restorers across three environments.

Parameters	DFF	PH	PL	PER	PTN	SF	FG	TSW	SYP
Mean	98.19	104.23	25.37	104.61	11.42	84.3	161.38	21.53	22.35
SE	0.21	0.31	0.06	0.29	0.08	0.2	0.98	0.09	0.22
SD	8.01	11.72	2.36	10.92	3.21	7.4	37.25	3.46	8.16
Min	74 (RR177, E2)	76.6 (RR183,E3)	17.33 (RR180,E3)	71.11 (RR22, E3)	4.33 (RR216,E3)	47.76 (RR210, E3)	74 (RR82, E1)	9.04 (RR224, E3)	5.16 (RR215, E2)
Max	122 (RR110,E2)	140 (RR54,E1)	31.6 (RR39,E1)	135.64(RR132, E3)	33 (RR240,E2)	94.33 (RR123, E3)	410 (RR103,E3)	32.1 (RR86, E2)	60 (RR121, E1)
Min ENV	E2 (97.14)	E2 (101.82)	E3 (24.41)	E3 (101.21)	E3 (9.77)	E1 (83.97)	E2 (153.5)	E2 (20.29)	E2 (15.54)
Max ENV	E3 (98.78)	E1 (107.58)	E1 (26.5)	E2 (108.78)	E2 (13.8)	E3 (84.79)	E3 (171.72)	E1 (22.8)	E1 (29.36)
Min GEN	RR143 (83.83)	RR182 (86.07)	RR180 (21.12)	RR103 (89.44)	RR205 (8.28)	RR210(67.72)	RR215 (101.08)	RR99 (15.85)	RR215 (12.03)
Max GEN	RR228 (113.17)	RR54 (127.77)	RR39 (28.55)	RR177 (142.18)	RR86 (16.5)	RR165 (92.44)	RR99 (234.11)	RR86 (32.1)	RR121 (37.43)
*σ* ^2p^	64.38	133.65	5.53	118.94	10.27	54.82	1329.06	10.27	65.69
*σ* ^2g^	34.92	57.90	1.34	34.50	0.65	10.58	383.30	0.65	0.98
*h*^2bs^ (%)	81.22	81.33	66.87	61.17	29.03	34.06	45.35	64.06	51.15
GEIr^2^	0.61	0.08	0.87	0.25	0.09	0.37	0.48	0.55	0.21
AS	0.53	0.91	0.63	0.8	0.83	0.57	0.80	0.69	0.90
*r*_ge_	0.67	0.15	0.87	0.34	0.13	0.42	0.72	0.69	0.42
CVg	8.11	7.31	3.72	5.3	4.69	7.42	7.57	10.00	5.80
CVr	14.10	7.13	3.18	6.8	6.67	17.06	5.62	10.83	4.33
CV ratio	0.58	1.03	1.16	0.78	0.70	0.44	1.35	0.92	1.34
LRT_ge_	120.00	13.70	9.54	75.51	115.00	145.00	416.00	464.00	384.00
*P*-value	<0.001 ***	<0.001 ***	<0.001 ***	<0.001 ***	<0.001 ***	<0.001 ***	<0.001 ***	<0.001 ***	<0.001 ***

Genotype (GEN) and genotype × environment interaction (G×E) effects were tested using likelihood ratio tests.

DFF, days to 50% flowering (days); PH, plant height (cm); PL, panicle length (cm); PER, panicle exertion ratio; SF, spikelet fertility (%); PTN, productive tiller number; TSW, thousand seed weight (g), SYP, seed yield per plant (g); SE, standard error; SD, standard deviation; CV, coefficient of variation; *σ*^2p^, phenotypic variance; *σ*^2g^, genotypic variance; *σ*^2ge^, GEI variance; *h*^2bs^ (%), broad sense heritability in percentage; GEIr^2^, GEI coefficient of determination; AS, accuracy of genotype selection; *r*_ge_, association among genotypic values across environments; CVg, genotypic coefficient of variation; CVr, residual coefficient of variation; LRT_ge_, likelihood ratio test for GE interaction; *P*-value, probability value. ***P < 0.001.

### 3.3 Variance components and likelihood ratio test

Likelihood ratio tests revealed significant genotypic effects for all traits, indicating substantial genetic variability among the rice restorers. Genotype × environment interactions were highly significant (*P* < 0.001) for all traits, except PL which is significant at *P* < 0.01, demonstrating the differential behavior of genotypes with changes in the environment. Phenotypic variance (*σ*²_p_) was highest for FG (1329.06), followed by PH (133.65), PER (118.94), SYP (65.69), and DFF (64.38), whereas genotypic variance (*σ*²_g_) was highest for FG (383.30), PH (57.90), PER (34.50), and DFF (34.92). Broad-sense heritability (*h*²_bs_) estimates were high for PH (81.33%) and DFF (81.22%), moderate to high for PL (66.87%), TSW (64.06%), and PER (61.17%), moderate for SYP (51.15%) and FG (45.35%), and low for SF (34.06%) and PTN (29.03%).

The proportion of variance due to genotype × environment interaction (GEIr²) was highest for PL (0.87), followed by DFF (0.61), TSW (0.55), FG (0.48), SF (0.37), PER (0.25), and SYP (0.21), while PH (0.08) and PTN (0.09) exhibited relatively lower GEI contributions. The selection accuracy (AS) values ranged from 0.53 for DFF to 0.91 for PH, with high accuracy observed for PH (0.91), SYP (0.90), PTN (0.83), PER (0.80), FG (0.80), and TSW (0.69). Genotypic correlations across environments (*r*_ge_) were high for PL (0.87), FG (0.72), TSW (0.69), and DFF (0.67), moderate for SYP (0.42), SF (0.42), and PER (0.34), and low for PH (0.15) and PTN (0.13), indicating a trait-specific consistency of restorer’s performance across environments. The genetic coefficients of variation (CV_g_) ranged from 3.72% for PL to 10.00% for TSW, while the residual coefficients of variation (CV_r_) varied from 3.18% for PL to 17.06% for SF. The CV ratio exceeded unity for PL (1.16), FG (1.35), and SYP (1.34), indicating greater genetic variability for these traits than for the other traits. Likelihood ratio tests confirmed highly significant genotype × environment interactions for all traits, with LRTge values ranging from 9.54 (PL) to 464.00 (TSW) (*p* < 0.001) (**Table 3**).

### 3.4 Trait associations and distribution patterns across environments

The pairwise correlation matrix revealed distinct patterns of association among morphological and yield-related traits in the three environments. The diagonal density plots indicated that most traits followed near-normal distributions with environment-specific shifts in their central tendency, highlighting the differential environmental influence on the trait’s performance (**Figure 1B**). DFF exhibited a positive and significant association with PH (0.266) and PL (0.212) while negative correlations with PTN (-0.117) and SF (-0.056). PH showed a strong positive correlation with PL (0.395) and moderate positive associations with SYP (0.182) and FG (0.111), respectively. In contrast, PH was negatively correlated with SF (-0.057) and PTN (-0.046), while its association with PER was weak and non-significant (-0.024). SF showed a negative association PER (-0.114) while maintaining positive correlations with PL (0.033) and SYP (0.090). SF showed a positive correlation with PL (0.033) and SYP (0.090). PL was positively correlated with PH (0.395) and SYP (0.176) while negatively associated with PTN (-0.091). PTN exhibited a negatively significant relationship with DFF (-0.117), PER (-0.204), FG (-0.105), and SYP (-0.131). TSW showed a significant positive correlation with SYP (0.336), PL (0.095), PER (0.063), and PH (0.098) and a significant negative association with PTN (-0.091).

### 3.5 BLUP-based stability analysis and ranking of rice restorers using WAASB and WAASBY indices

**Figure 2:**
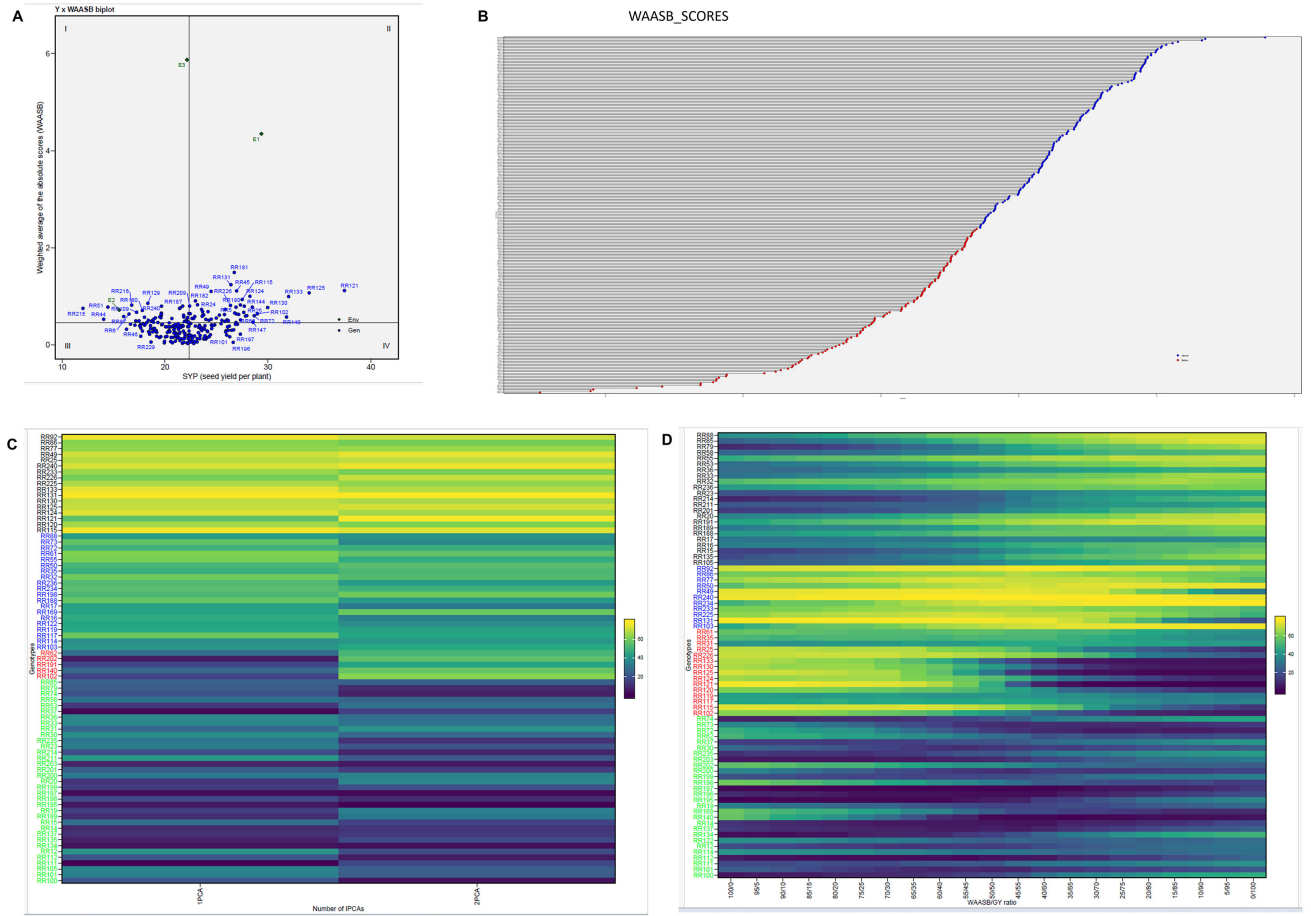
**(A)** WAASB × seed yield per plant **(Y)** biplot for the 240 rice restorers in three environments. **(B)** Ranking of genotypes using the WAASBY index for the simultaneous selection of grain yield and stability. **(C)** Genotype clustering into four groups based on the number of principal component axes utilized to calculate the WAASB index. **(D)** Genotype clustering into four groups based on WAASB/GY ratio.

In the present study, the *Q*–*Q* plots showed that most points closely follow the theoretical quantile line, with only minor deviations at the tails (**Figure 1C**), which indicates that the distribution of various BLUP effects for BLUPbre is normal and for BLUPg, BLUPge, and BLUPg+ge+bre close to normal. This indicates that the assumption of normality for random effects was largely met. The overall mean WAASBY value across the 240 restorers was 57.15, with 129 restorers above and 111 restorers below the WAASBY mean values, respectively. Based on WAASBY ranking, the top 10% of restorers (24 restorers) were selected as superior restorers (**Figure 2B**) (**Supplementary Table 4**). Among the 24, the restorers RR196 (77.9), RR197 (73.5), RR101(73.3), RR137 (71.5), and RR100 (71.1) were the top five restorers based on the WAASBY value. To further refine the selection, the WAASBY results were integrated with the BLUP-derived mean yield (Y) × WAASB biplot, which classifies genotypes into four quadrants using the mean yield and WAASB (**Figure 2A**). Quadrant I contained restorers such as RR51 (0.779), RR65 (0.80), and RR129 (0.86), with low yield and high WAASB, representing highly unstable and undesirable lines. Quadrant II included restorers RR45 (1.11), RR49 (1.10), RR121 (1.12), RR125 (1.07), and RR131(1.24), with high yield but high WAASB, indicating specific adaptability and poor stability across locations. Quadrant III comprised restorers RR46 (0.323), RR50 (0.481), RR218 (0.296), and RR232 (0.179), which are low-yielding yet stable (low WAASB values), which may have limited use unless targeted for specific stress-related environments. Restorers present in quadrant IV, RR23 (0.060), RR100 (0.029), RR105 (0.084), RR196 (0.053), and RR214 (0.080), are characterized by above-average yield and below-average WAASB values and considered ideal due to their high performance coupled with greater stability across environments.

Among the environments, E2 showed lower WAASB values, suggesting that it was more representative, whereas E1 and E3 were more discriminative. The first heatmap (**Figure 2C**) illustrates restorer clustering based on the number of PCs utilized to calculate the WAASB index. The second heatmap (**Figure 2D**) shows restorer clustering according to the WAASB/SYP ratio. When using a ratio of 100/0, the rankings are determined solely by stability, while a ratio of 0/100 focuses exclusively on productivity. In both plots, the restorers are clustered into four groups: red for unproductive and unstable, blue for productive but unstable, black for stable but unproductive, and green for productive and stable.

### 3.6 Genotype selection across the test environments using different selection indices

A total of 240 rice restorers were evaluated in three different locations. These rice restorers were further analyzed using MGIDI, MTSI, MTMPS, and FAI-BLUP to identify the most stable and high-performing rice restorers and to compare the efficiency of these indices.

#### 3.6.1 Multi-trait genotype–ideotype distance index

##### 3.6.1.1 Loadings and factor description for MGIDI

Applying the Kaiser criterion, which retains factors with eigenvalues exceeding 1, revealed four factors that together account for 65.4% of the total variance in the dataset as assessed by the WAASBY value of BLUP estimates. The eigenvalues, explained variance, and factorial loadings from MGIDI factor analysis are summarized in **Table 4**. The first factor (FA1) had an eigenvalue of 2.18 and accounted for 24.2% of the variance. The second factor (FA2) had an eigenvalue of 1.49, explaining 16.6% of the variance, while the third factor (FA3) and fourth factor (FA4) showed an eigenvalue of 1.18 and 1.03, accounting for 13.1% and 11.4% of the total variance, respectively. FA1 is primarily associated with DFF (-0.68), PH (-0.81), and PL (0.8). FA2 is strongly linked to PTN (0.62), TSW (0.67), and FG (-0.67). FA3 is contributed by SF (-0.72) and SYP (-0.78). Finally, the fourth factor is associated with PER (-0.96). After varimax rotation, the average communality (*h*) was 0.65, with communalities for the traits ranging from 0.48 (PTN) to 0.93 (PER), indicating the proportion of each trait’s variance accounted for by the extracted factors. Uniqueness values range from 0.07 to 0.52, representing the variance in each trait not explained by the factors (**Table 4**).

**Table 4:** Factor analysis results from MGIDI evaluation of grain yield and its attributes across the three environments.

VAR	FA1	FA2	FA3	FA4	Communality	Uniquenesses
SYP	0.14	0.33	-0.78	0.07	0.74	0.26
PH	-0.81	-0.02	-0.04	-0.11	0.67	0.33
PER	-0.03	0.04	-0.01	-0.96	0.93	0.07
SF	-0.32	-0.09	-0.72	-0.09	0.64	0.36
PL	0.8	0	0.16	-0.03	0.66	0.34
PTN	-0.17	0.62	-0.21	0.14	0.48	0.52
TSW	0.19	0.67	-0.2	-0.23	0.58	0.42
FG	0.28	-0.67	-0.33	0.02	0.63	0.37
DFF	-0.68	0.27	0.07	0.07	0.55	0.45
Eigen values	2.18	1.49	1.18	1.03	–	–
Variance, %	24.2	16.6	13.1	11.4	–	–
Cumulative variance, %	24.2	40.8	53.9	65.4	–	–

VAR, variable or trait; FA1, factor 1; FA2, factor 2; FA3, factor 3, DFF, days to 50% flowering (days); PH, plant height (cm); PL, panicle length (cm); PER, panicle exertion ratio; SF, spikelet fertility (%); PTN, productive tiller number; TSW, thousand seed weight (g); FG, filled grains per panicle; SYP, seed yield per plant (g).

#### 3.6.2 Genotype selection and selection gain based on MGIDI analysis

**Figure 3:**
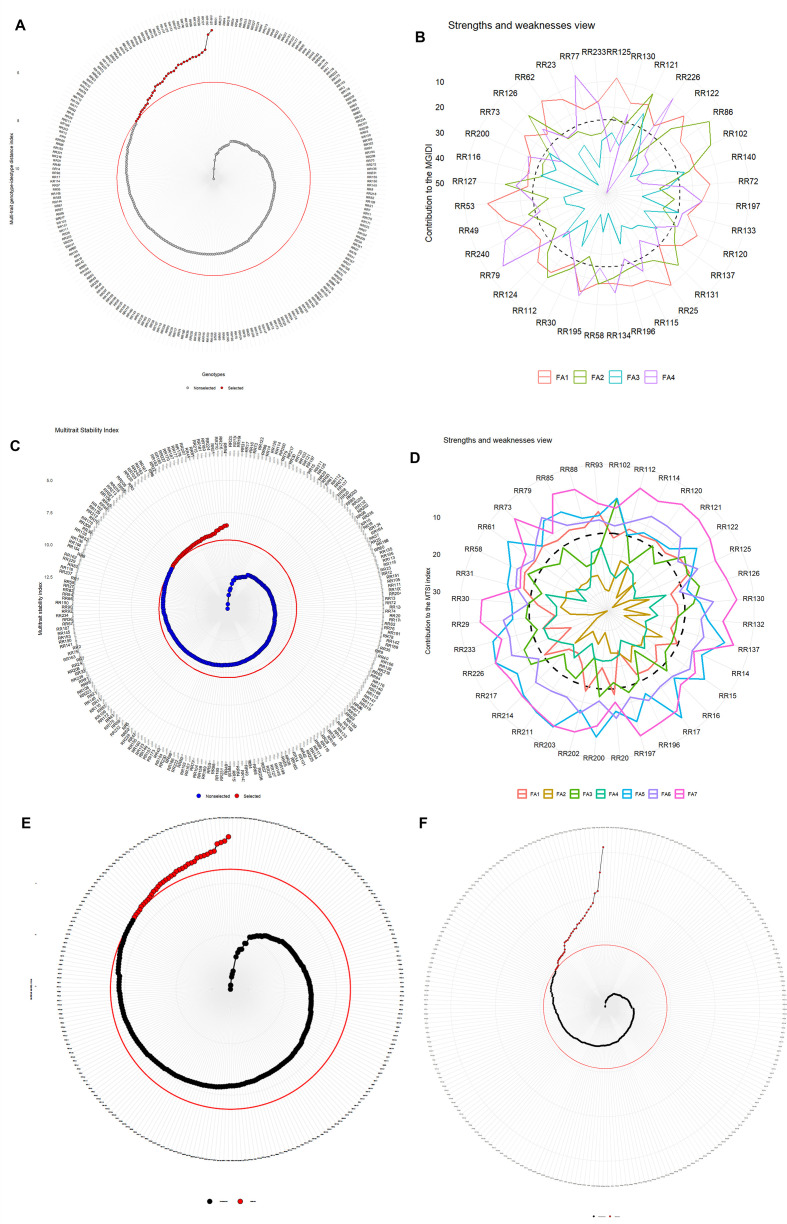
**(A)** Genotype ranking in ascending order for the MGIDI index tested under multi-environmental trials with selection intensity of 15% (red circle). The selected genotypes are highlighted in red. The scale in the radar plot represents the MGIDI score. The red circle represents the threshold MGIDI score, the inner circle next to the threshold circle represents the smallest value of the scale, and the innermost circle represents the highest value of the scale. **(B)** Strength and weakness view of the stable genotypes identified across three environments, shown as the proportion of each factor on the computed MGIDI index. **(C)** Genotype ranking in ascending order for the MTSI index tested under multi-environmental trials with selection intensity of 15% (red circle). **(D)** Strength and weakness view of the stable genotypes identified across three environments, shown as the proportion of each factor on the computed MTSI index. **(E)** Selection of rice restorers at 15% selection intensity using MTMPS. **(F)** Selection of rice restorers at 15% selection intensity using FAI_BLUP.

The genotype ranking, determined by the MGIDI score, is presented in **Figure 3A**. From the 240 rice restorers, 36 rice restorers were selected based on 15% selection intensity. The selected 36 rice restorers are given in **Supplementary Table 5**.

#### 3.6.3 Strengths’ and weaknesses’ view

**Figure 3B** illustrates the strengths and weaknesses of the selected 36 rice restorers from 240 rice restorers as determined by each factor’s proportional contribution to the genotypes MGIDI scores. The strengths’ and weakness’s view reveal how much each factor (FA1–FA4) contributes to the MGIDI index of each selected genotype. These factors are obtained by factor analysis, where correlated traits are grouped together. The MGIDI method identifies genotypes closest to the ideotype and highlights trait-groups contributing most to the deviation. The distance from the center represents the contribution of each factor to the MGIDI value. Lower contribution values (closer to the dashed circle) indicate strengths, whereas higher contributions (peaks away from the center) indicate weaknesses. The dashed circle shows the average MGIDI contribution across genotypes. FA1 is mainly associated with yield attributes like PH, PL, and DFF. Restorers which are showing low FA1 contribution (closer to the dashed circle) demonstrate strong performance for these associated traits. Restorers such as RR125, RR130, RR121, RR102, RR140, and RR226 show FA1 as a strength. Restorers like RR116, RR53, and RR49 show a high FA1 contribution, which means weakness for yield-related traits. FA2 is with PTN, TSW, and FG; rice restorers like RR102, RR122, and RR226 display low FA2 contribution and, hence, large panicle and grain performance. Restorers such as RR73, RR200, and RR124 show high FA2 contribution, indicating a weaker performance in FA2-associated traits. The rice restorers RR130, RR131, RR197, and RR120 show FA3 as a strength for traits like SF and SYP. FA4 is associated with trait PER, while restorers like RR122, RR131, and RR226 show FA4 as strength, implying desirable PER.

#### 3.6.4 Predicted selection differential gain obtained through MGIDI

**Figure 4:**
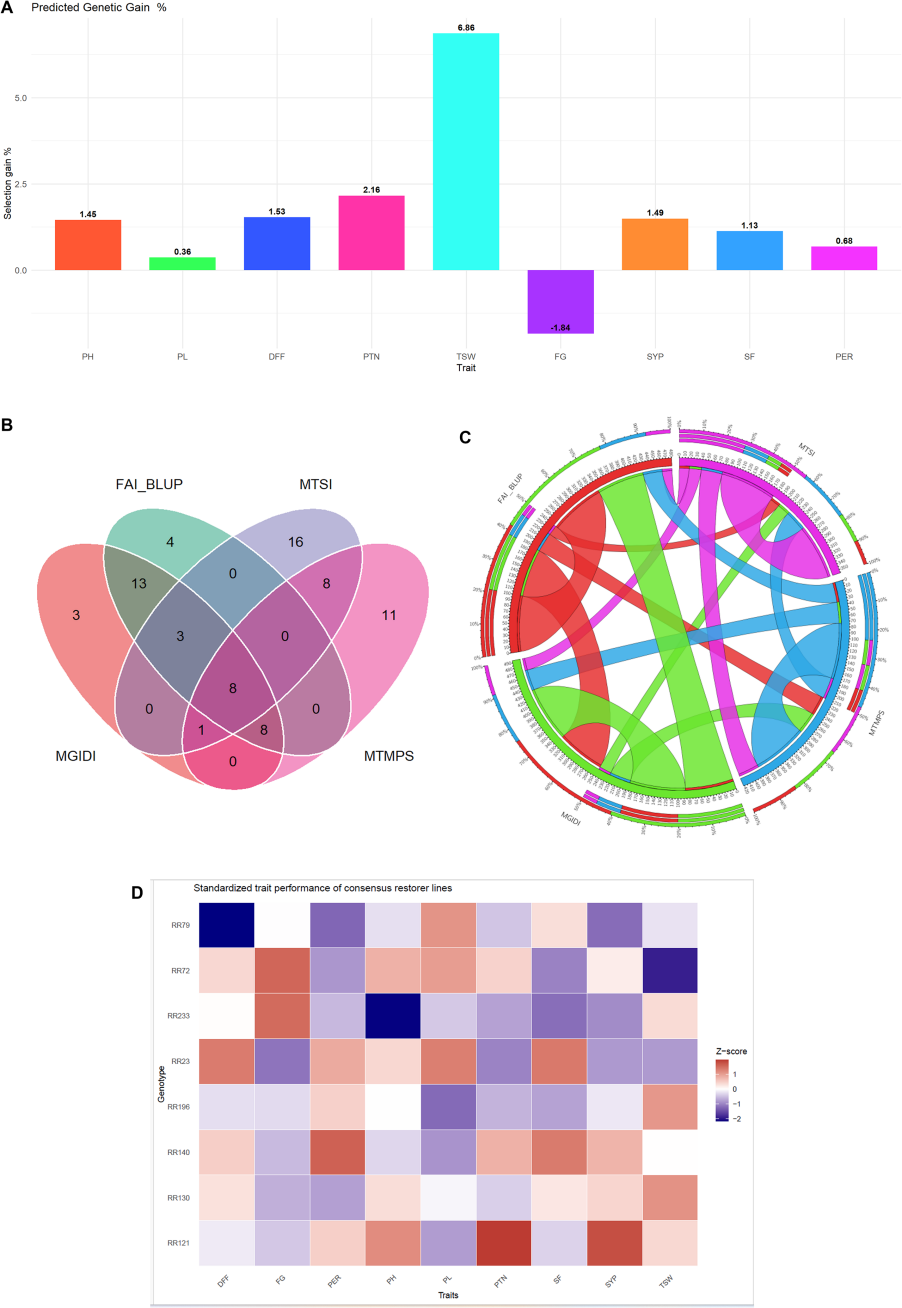
**(A)** Percentage of predicted selection differential gain achieved in multi-environment trials through MGIDI. **(B)** Venn diagrams showing the number of common rice restorers selected across models. **(C)** Circos plot representing the coincidence index among the various multi-trait stability models. **(D)** Standardized trait performance (Z-scores) of eight consensus rice restorer lines identified by MGIDI, MTSI, MTMPS, and FAI-BLUP indices. Z-scores were calculated within the selected genotype set, illustrating relative trait strengths and trade-offs among elite restorers. Red and blue colors indicate positive and negative standardized deviations from the trait mean, respectively.

The predicted genetic gains under selection and selection differentials for WAASB of all the traits are presented in **Figure 4A**. The MGIDI index achieved favorable selection differential for eight out of nine traits. Positive selection differentials were observed for all of the traits, except FG (-1.84). The percent of selection differential is ranging from -1.84 for FG to 6.86 for TSW. The genetic gain under selection (ΔG%) is ranging from -0.824 for the trait FG to 2.91 for the trait TSW. These results reflect the differential responses of traits to selection pressure and provide insights for targeted breeding strategies (**Table 1**).

### 3.7 Coincidence index, common genotypes selected from the different multi-trait-based stability models

In addition to MGIDI, several other multi-trait stability models were employed to select rice restorers with 15% selection intensity (**Supplementary Table 5**). The restorers selected based on the MTSI (**Figures 3C, D**), MTMPS (**Figure 3E**), and the FAI-BLUP (**Figure 3F**) methods are illustrated in **Figures 3B–D**, respectively. A comparison of these models is illustrated through a Venn diagram representing (**Figure 4B**) the common restorers across the different models and coincidence index through the Circos plot (**Figure 4C**). The coincidence index between the models will be higher when more restorers are identified between the models. The maximum coincidence index was observed between MGIDI and FAI_BLUP (86.92%), with 32 common rice restorers, followed by MGIDI and MTMPS (37.90%, 17 common restorers), MTSI and MTMPS (37.90%, 17 common restorers), FAI_BLUP and MTMPS (34.64%, 16 common restorers), and MGIDI and MTSI (21.57%, 12 common restorers). The minimum coincidence index was observed between FAI_BLUP and MTSI (18.30%, 11 common restorers). In our present study, eight rice restorers (RR130, RR121, RR72, RR140, RR196, RR79, RR23, and RR223) were consistently identified across all four selection models, demonstrating a strong agreement among the different multi-trait selection approaches (**Table 5**).

**Table 5:** List of common rice restorers identified across the different multi-trait selection indices.

SN	Methods	Count	Name of the genotypes
1	MGIDI and FAI_BLUP	32	RR125, RR130, RR226, RR121, RR72, RR122, RR197, RR140, RR133, RR120, RR102, RR25, RR137, RR115, RR86, RR195, RR196, RR53, RR134, RR131, RR79, RR58, RR112, RR124, RR30, RR49, RR240, RR62, RR23, RR233, RR77, RR211
2	MGIDI and MTSI	12	RR130, RR121, RR72, RR140, RR137, RR86, RR196, RR79, RR73, RR23, RR233, RR211
3	MGIDI and MTMPS	17	RR125, RR130, RR121, RR72, RR122, RR140, RR120, RR102, RR195, RR196, RR134, RR79, RR58, RR112, RR73, RR23, RR233
4	FAI_BLUP and MTSI	11	RR130, RR86, RR121, RR72, RR140, RR137, RR196, RR79, RR233, RR211, RR23
5	FAI_BLUP and MTMPS	16	RR125, RR130, RR121, RR122, RR102, RR72, RR120, RR140, RR195, RR134, RR196, RR112, RR79, RR58, RR233, RR23
6	MTSI and MTMPS	17	RR17, RR196, RR72, RR23, RR140, RR79, RR36, RR203, RR73, RR114, RR14, RR111, RR32, RR121, RR130, RR198, RR233
7	MGIDI, MTMPS, MTSI and FAI_BLUP	8	RR130, RR121, RR72, RR140, RR196, RR79, RR23, RR233

### 3.8 Comparative multi-trait profiling of consensus elite rice restorer lines

Standardized multi-trait profiling was performed for the eight consensus restorer lines that were consistently identified across MGIDI, MTSI, MTMPS, and FAI-BLUP selection frameworks (**Figure 4D**). Trait values were standardized using *Z*-scores calculated within this consensus set to facilitate a relative comparison among elite restorers. The heatmap revealed a clear, trait-wise performance differentiation, highlighting both strengths and trade-offs among restorers. Restorers RR121 and RR130 exhibited superior standardized values for seed yield per plant (SYP) along with favorable performance for panicle traits, indicating their strong yield potential. RR140 and RR196 showed a balanced performance across multiple yield-contributing traits, particularly spikelet fertility (SF), filled grains per panicle (FG), and panicle length (PL), suggesting robust agronomic suitability. RR72 and RR79 were characterized by favorable early to mid-early flowering (lower DFF) combined with moderate yield attributes, making them desirable for maturity diversification in hybrid breeding programs. Overall, the standardized heatmap emphasizes trait complementarity among the consensus restorers rather than absolute superiority, reinforcing the effectiveness of multi-trait, multi-environment selection indices in identifying genetically diverse yet agronomically elite parental lines suitable for hybrid rice breeding.

## 4 Discussion

The present study aimed at identifying and grouping of 240 diverse restorer lines based on allelic status of *Rf_3_* and *Rf_4_* genes and then evaluating restorer lines across the three diverse environments. The study focused on the main traits such as DFF, PH, PTN, PER, SF, PL, TSW, FG, and SYP. These traits are crucial for identifying the early-duration restorers that possess other favorable combinations of traits suitable for future hybrid breeding programs. To develop rice hybrids that can consistently mature early across various environments, it is important to study how the parents of hybrids interact with the environment. This understanding was emphasized by GEI (Comstock and Moll, 1963). In light of this, our study employed marker-assisted selection of restorer lines based on the *Rf_3_* and *Rf_4_* genes and advanced multivariate techniques like MGIDI, MTSI, MTMPS, and FAI_BLUP for the selection of rice restorers which are stable, high-performing, and superior for other agronomic traits.

Molecular-marker-based characterization of 240 diverse restorer lines showed that 3.34% possessed the *Rf_3_* restorer allele, while 85.41% possessed the *Rf_4_* restorer allele. This indicates the predominance of the *Rf_4_* restorer allele in the Indian germplasm. This is in agreement with earlier studies of Katara et al. (2017). He reported the predominance of *Rf_4_* allele in Indian germplasm, wherein 19% of the varieties had *Rf_3_* and 63% of the varieties carried the *Rf_4_* gene. It is well known that the wild abortive (WA) fertility restorer genes in *japonica* are less frequent than in the *indica* gene pool (Lin and Yuan, 1980; Siddiq and Singh, 2005; Virmani and Edwards, 1983). The current study consists of only eight lines positive for *Rf_3_* alone, and a significant difference between *Rf_3_* and *Rf_3_Rf_4_* was observed for SF (%). There is an increase in the trend for SF (%). This is because *Rf_4_* is a predominant gene to restore the fertility against WA cytoplasm, and *Rf_3_* acts as a synergistic gene (Shidenur et al., 2020). RM6100 is a gene-linked marker (*Rf_4_*), rather than gene-based, and therefore carries an inherent risk of marker–gene recombination, which could potentially result in false-positive or false-negative classifications (Virmani et al., 2003; Li et al., 2016). However, the selected markers have been extensively validated and widely adopted in rice hybrid breeding programs due to their close linkage with major fertility restoration loci and their practical utility in large-scale screening (Hu, 2012; Huang et al., 2014; Kumar et al., 2017b). In operational breeding pipelines, such linked markers are routinely employed as a first-tier enrichment tool to identify restorer candidates, with the understanding that the final confirmation of restoration ability requires phenotypic validation through CMS-based test-crosses (Virmani and Kumar, 2004; Li et al., 2016). A substantial proportion of the material used in this study (198 out of 240 lines) consists of iso-cytoplasmic restorer lines (WA-CMS). The iso-cytoplasmic restorer lines are unique as they carry the same male sterile cytoplasm as the cytoplasmic male sterile (CMS) lines, which minimizes the potential cyto-nuclear conflict between the cytoplasmic and nuclear genes. Additionally, the range of SF (%) data of restorer lines carrying *Rf_3_*, *Rf_4_*, and the combination of *Rf_3_* & *Rf_4_* corresponds with previous reports (Kumar et al., 2017b; Shidenur et al., 2019). Moreover, the current study aims to study the stability of the restorer lines and identify the best restorer lines based on the per se performance of the line supplemented by the fertility restorer gene data. Therefore, the likelihood of recombination between linked markers and target *Rf* loci affecting classification accuracy in this panel is expected to be limited (Virmani et al., 2003; Huang et al., 2014).

The pooled analysis of variance revealed highly significant (*P* < 0.001) effects of environments, genotypes, and GEI for all yield and yield-attributing traits. The presence of significant GEI for complex traits such as SYP, FG, and PER indicates differential genotype responses across environments, which is a common feature in rice multi-location trials and reflects environmental modulation of genetic expression (Yan and Kang, 2003; Crossa et al., 2017). Such crossover and non-crossover interactions confound direct phenotypic selection and reduce the efficiency of genotype identification when selection is based solely on mean performance. The magnitude of the GEI variance, as further supported by high LRT values and significant *p*-values across traits, demonstrates that genotype rankings are not stable and no single trait provides a reliable basis for selection across environments. Under these conditions, conventional univariate selection or yield-only indices tend to favor genotypes with specific adaptation rather than broad adaptability (Gauch, 2013). Favorable CVg/CVr ratios observed for SYP, PH, FG, and PTN indicate a predominance of exploitable genetic variation, supporting their inclusion as key drivers in multi-trait selection frameworks rather than as independent selection targets. Conversely, traits like PER and SF were characterized by strong GEI (high GEIr²) and lower heritability, rendering them unsuitable for direct selection but still relevant for overall agronomic adaptation (Piepho et al., 2012). These patterns together justify the adoption of multi-environment mixed-model and multi-trait selection indices that jointly exploit BLUP-based genetic predictions while accounting for GEI, thereby improving the efficiency and robustness of genotype selection. The correlation analysis across environments revealed moderate to strong associations among yield and yield-related traits. Particularly between SYP and FG, TSW, PL, and PH, such inter-trait relationships are well reported in rice (Yoshida, 1981; Peng et al., 2008; Kumar et al., 2025). However, the presence of correlated traits also indicates multicollinearity, which can bias selection decisions by disproportionately weighting redundant traits when conventional index-based or univariate approaches are applied (Graham, 2003; Hair et al., 2019). This issue is further exacerbated under multi-environment testing, as correlated traits often exhibit differential GEI patterns, reducing the reliability of trait-wise selection across locations (Yan and Kang, 2003; Crossa et al., 2017).

Multi-trait multi-environment selection indices such as MGIDI, MTSI, MTMPS, and FAI-BLUP explicitly address these challenges by incorporating trait covariance and GEI information into genotype ranking. The MGIDI index, in particular, employs factor analysis to group correlated traits into latent factors, thereby reducing multicollinearity and preventing the over-representation of highly correlated yield components such as FG, TSW, and PL (Olivoto and Nardino, 2021). Similarly, MTSI and MTMPS integrate multi-trait BLUPs with stability metrics, allowing genotypes to be selected based on their overall performance and adaptability across environments while accounting for trait interdependence (Olivoto et al., 2019b; Rocha et al., 2021). The FAI-BLUP index further refines selection by combining factor scores with ideotype-based distance measures, ensuring balanced improvement across correlated traits under multi-environment conditions (Olivoto and Nardino, 2021). The high coincidence observed among MGIDI, MTSI, MTMPS, and FAI-BLUP rankings in the present study indicates that these indices consistently exploit the underlying correlation structure while maintaining selection stability across environments. Overall, the observed correlation patterns among yield and its component traits, coupled with significant GEI effects, justify the adoption of multi-trait multi-environment indices over conventional correlation-based or single-trait selection methods. By effectively handling multicollinearity and integrating GEI, these indices provide a genetically sound framework to identify stable and superior rice restorers, which is essential for their subsequent utilization in hybrid rice breeding programs.

The identification of superior genotypes in multi-environment trials requires a simultaneous consideration of mean performance and yield stability, as selection based solely on mean yield often leads to the advancement of genotypes with poor adaptability across environments (Piepho et al., 2008; Smith et al., 2005). In the present study, the WAASBY index effectively integrated BLUP-based grain yield and stability (WAASB), enabling a balanced selection of genotypes that combined high productivity with consistent performance across environments (Olivoto et al., 2019; Olivoto and Nardino, 2021; Danakumara et al., 2023). Such an integrated approach is particularly relevant in hybrid breeding programs, where the stability of parental lines is essential to ensure a predictable hybrid performance across diverse target environments (Hallauer et al., 2010). All 24 restorers selected under 10% selection intensity based on WAASBY also belonged to the top restorers of quadrant IV of the Y × WAASB framework. Quadrant IV genotypes are widely recognized as ideal in stability analyses, as they express superior mean performance while exhibiting reduced genotype × environment interaction effects, indicating broad adaptation and environmental resilience (Crossa et al., 2011; Yan and Kang, 2003). This complete overlap between index-based selection and quadrant-based classification provides strong evidence of the robustness and reliability of the selection strategy (Olivoto et al., 2019a; Yan and Kang, 2003), but the quadrant-based classification is conditional on the three environments studied, and including more years would further strengthen the stability inference.

From a plant breeding perspective, the fact that all top-ranked genotypes under stringent selection intensity also satisfied the most desirable stability criterion highlights their potential value as elite restorer lines. Such genotypes are particularly suitable for hybrid breeding, where stable parental performance across environments is critical to maintain a heterotic expression and reduce environmental risk (Hallauer et al., 2010; Betran et al., 2003). The integration of WAASBY-based stability analysis with multi-trait and multi-environment selection models (MGIDI, MTSI, MTMPS, and FAI-BLUP) therefore provides a comprehensive and biologically sound framework to identify restorers with high breeding value and wide adaptation (Rocha et al., 2018, 2021; Olivoto and Nardino, 2021; Dudhe et al., 2024). We further note that WAASB- and WAASBY-based metrics are designed to provide robust genotype ranking under limited but contrasting environment sets by decomposing genotype × environment interaction using BLUPs. When complemented by coincidence analysis across multiple multi-trait and stability indices, this approach reduces environment-specific bias and enhances confidence in genotype identification. Nevertheless, we explicitly acknowledge that multi-year testing would further strengthen inference on long-term adaptation, and such evaluation is proposed as a subsequent step at the hybrid validation stage.

Factor analysis partitioned the nine correlated traits into four independent factors that together explain 65.4% of the total phenotypic variance, indicating an effective summarization of the multivariate trait structure and supporting the application of factor-based multi-trait selection (Rocha et al., 2018; Olivoto and Nardino, 2021). The first factor (FA1), accounting for a large variance of 24.2% of the variance, was primarily associated with PH, PL, and DFF. This factor separates tall, late restorer lines from shorter and mid-early. In rice, these traits are highly correlated and often occurs together in multivariate analyses (Akinwale et al., 2011; Chandra et al., 2020). This factor is therefore critical for breeding ideotypes with improved plant architecture and lodging tolerance. Defining the ideotype toward moderate plant height and earliness along FA1 enables the MGIDI index to penalize very tall or very late genotypes even if they have good yields. FA3, which explains 13.1% of the variance, was associated with SYP and SF. Grouping SYP and SF within the same factor allows MGIDI to favor genotypes combining high spikelet fertility with superior grain yield potential. FA4 explained 11.4% of the variance and was almost entirely defined by PER with a very high communality 0.93. This indicates that PER behaves as a relatively independent trait. Poor exertion is known to reduce spikelet fertility and affects efficient outcrossing in hybrid rice seed production (Virmani and Kumar, 2004). Treating PER as a separate factor (FA4) ensures that MGIDI explicitly penalizes restorers with low panicle exertion, even when their yield components are favorable.

The coincidence analysis revealed the consistency in the selection of restorers across different stability models, underscoring the robustness of certain restorer lines in the study. Specifically, the eight restorers—RR130, RR121, RR72, RR140, RR196, RR79, RR23, and RR233—were common across the models, and also these eight restorers among the 24 restorers selected at 10% selection intensity based on WAASBY were also present in quadrant IV of the Y × WAASB framework, highlighting their stability and potential for favorable agronomic performance. The selection of unique restorer lines in each model highlighted the importance of using multiple models to obtain a comprehensive view of genotype performance and stability. Similarly, Raj et al. (2024) utilized MTSI and MGIDI in cotton to identify early-maturing ideotypes suitable for various planting densities. Benakanahalli et al. (2021) and Ambrosio et al. (2024) respectively employed MGIDI and FAI-BLUP in in guar and black beans for screening genotypes across multiple environments.

The majority of plant breeders have applied classic stability indices such as mean, regression, and deviation from regression parameters to choose stable genotypes. The AMMI and GGE biplot analysis are helpful to breeders in the identification of mega-environments for selecting and discriminating test environments (Yan and Hunt, 2001). The GGE biplot is a widespread methodology used to analyze MET data for the evaluation of genotype and test environment vis-à-vis the interpretation of complex GEI interactions (Yan and Tinker, 2006). However, these statistical tools were insufficient due to their reliance on fixed effects, susceptibility to collinearity and outlier issues, and challenges in visualizing complex interactions to identify the strengths and weaknesses of genotypes and selecting those with the desired mean performance and stability (Bhering et al., 2012). Multiple trait selection indices, such as the MTSI and MGIDI evaluation systems, were found to be novel and unique techniques in plant breeding practices (Abdelghany et al., 2021; Benakanahalli et al., 2021; Hadou El Hadj et al., 2022; Yue et al., 2022; Mezzomo et al., 2023; Singamsetti et al., 2023). Thus, the MTSI and MGIDI methods have proven to be robust tools to identify genotypes with favored average performance and desired specific traits as well as to evaluate the strengths and weaknesses of selected and unselected genotypes without the issue of multicollinearity. This process maintains the original correlation structure of the data while simultaneously identifying superior genotypes based on multiple traits (Olivoto et al., 2019b). The multi-model-based selected restorer lines from this study will have proven wider adaptability that can be used to develop the hybrids adaptable to diverse geography. The practical breeding implications are addressed in the conclusion, where we explicitly state how the identified restorers can be utilized in CMS-based hybrid combinations, followed by multi-location testing of the derived hybrids. This clarifies how breeders can apply the proposed multi-trait, multi-environment selection framework to reduce selection risk and accelerate the development of stable, high-yielding hybrid rice cultivars. To our knowledge, no prior research has concurrently utilized MGIDI, MTSI, MTMPS, and FAI BLUP for the identification of rice restorer lines in multi-environment trials.

## 5 Conclusion

In the context of increasing climatic uncertainty and high genotype × environment interactions, the identification of restorers combining high productivity with stable performance across environments has become a central objective of modern plant breeding. This study demonstrates that relying on single-trait or conventional stability measures is inadequate for capturing the compound responses of genotypes under variable environments. The integration of BLUP-based stability analysis with multi-trait, multi-environment selection indices instead provides an efficient basis to identify broadly adapted restorer lines. From a hybrid breeding perspective, the identification of restorer lines having mid-early duration with stable performance in flowering and other yield-attributing traits is particularly important, as these attributes contribute to efficient fertility restoration, hybrid seed production, and consistent hybrid performance across target environments. Across all applied models, eight restorer lines (RR130, RR121, RR72, RR140, RR196, RR79, RR23, and RR233) consistently occurred as superior performers, and this minimizes the risk of model-dependent selection bias. These restorer lines have the favorable combination of early to mid-early flowering duration, high spikelet fertility, long well-exserted panicles, increased filled grain number, and high seed yield per plant coupled with relatively narrow phenotypic variation across environments, making them well suited for the development of high-yielding, climate-resilient, and widely adapted hybrids. Furthermore, the utilization of these identified restorers in CMS-based hybrid combinations, followed by multi-location evaluation of the derived hybrids, will facilitate the exploitation of heterotic potential and accelerate the deployment of next-generation hybrid rice cultivars under changing climatic conditions.

## Data Availability

The original contributions presented in the study are included in the article/**Supplementary Material**. Further inquiries can be directed to the corresponding author.
