# Supplementary material for: Integrating marker-assisted identification and multi-environment trait stability models to select superior rice restorer lines for hybrid breeding
Source: Front Plant Sci. 2026 Mar 11;17:1781490. doi: 10.3389/fpls.2026.1781490 (PMC13024654; doi:10.3389/fpls.2026.1781490)
Supplement: Supplementary file 1 [file Table1.docx]

Table S1 A set of 240 diverse restorers utilized in the study and their pedigree

| **SN** | **Lines** | **Pedigree** | **SN** | **Lines** | **Pedigree** | **SN** | **Lines** | **Pedigree** | **SN** | **Lines** | **Pedigree** |
| --- | --- | --- | --- | --- | --- | --- | --- | --- | --- | --- | --- |
| 1 | RR1 | CMH-1 | 61 | RR61 | CMH-63 | 121 | RR121 | CMH-124 | 181 | RR181 | MLT-44 |
| 2 | RR2 | CMH-2 | 62 | RR62 | CMH-64 | 122 | RR122 | CMH-125 | 182 | RR182 | MLT-45 |
| 3 | RR3 | CMH-3 | 63 | RR63 | CMH-65 | 123 | RR123 | CMH-126 | 183 | RR183 | MLT-46 |
| 4 | RR4 | CMH-4 | 64 | RR64 | CMH-66 | 124 | RR124 | CMH-127 | 184 | RR184 | MLT-47 |
| 5 | RR5 | CMH-5 | 65 | RR65 | CMH-67 | 125 | RR125 | CMH-128 | 185 | RR185 | MLT-48 |
| 6 | RR6 | CMH-6 | 66 | RR66 | CMH-68 | 126 | RR126 | CMH-129 | 186 | RR186 | MLT-49 |
| 7 | RR7 | CMH-7 | 67 | RR67 | CMH-69 | 127 | RR127 | CMH-130 | 187 | RR187 | MLT-50 |
| 8 | RR8 | CMH-8 | 68 | RR68 | CMH-70 | 128 | RR128 | CMH-131 | 188 | RR188 | MLT-51 |
| 9 | RR9 | CMH-9 | 69 | RR69 | CMH-71 | 129 | RR129 | CMH-132 | 189 | RR189 | MLT-52 |
| 10 | RR10 | CMH-10 | 70 | RR70 | CMH-72 | 130 | RR130 | CMH-133 | 190 | RR190 | MLT-53 |
| 11 | RR11 | CMH-11 | 71 | RR71 | CMH-73 | 131 | RR131 | CMH-134 | 191 | RR191 | MLT-54 |
| 12 | RR12 | CMH-12 | 72 | RR72 | CMH-74 | 132 | RR132 | CMH-135 | 192 | RR192 | MLT-55 |
| 13 | RR13 | CMH-13 | 73 | RR73 | CMH-75 | 133 | RR133 | CMH-136 | 193 | RR193 | MLT-56 |
| 14 | RR14 | CMH-14 | 74 | RR74 | CMH-76 | 134 | RR134 | CMH-137 | 194 | RR194 | MLT-57 |
| 15 | RR15 | CMH-15 | 75 | RR75 | CMH-77 | 135 | RR135 | CMH-138 | 195 | RR195 | MLT-58 |
| 16 | RR16 | CMH-16 | 76 | RR76 | CMH-78 | 136 | RR136 | CMH-139 | 196 | RR196 | MLT-59 |
| 17 | RR17 | CMH-17 | 77 | RR77 | CMH-79 | 137 | RR137 | CMH-140 | 197 | RR197 | MLT-60 |
| 18 | RR18 | CMH-18 | 78 | RR78 | CMH-80 | 138 | RR138 | MLT-1 | 198 | RR198 | MLT-61 |
| 19 | RR19 | CMH-19 | 79 | RR79 | CMH-81 | 139 | RR139 | MLT-2 | 199 | RR199 | PP-1 |
| 20 | RR20 | CMH-20 | 80 | RR80 | CMH-82 | 140 | RR140 | MLT-3 | 200 | RR200 | PP-2 |
| 21 | RR21 | CMH-21 | 81 | RR81 | CMH-83 | 141 | RR141 | MLT-4 | 201 | RR201 | PP-3 |
| 22 | RR22 | CMH-22 | 82 | RR82 | CMH-84 | 142 | RR142 | MLT-5 | 202 | RR202 | PP-4 |
| 23 | RR23 | CMH-23 | 83 | RR83 | CMH-85 | 143 | RR143 | MLT-6 | 203 | RR203 | PP-5 |
| 24 | RR24 | CMH-24 | 84 | RR84 | CMH-86 | 144 | RR144 | MLT-7 | 204 | RR204 | PP-7 |
| 25 | RR25 | CMH-25 | 85 | RR85 | CMH-87 | 145 | RR145 | MLT-8 | 205 | RR205 | PP-8 |
| 26 | RR26 | CMH-26 | 86 | RR86 | CMH-88 | 146 | RR146 | MLT-9 | 206 | RR206 | PP-9 |
| 27 | RR27 | CMH-27 | 87 | RR87 | CMH-89 | 147 | RR147 | MLT-10 | 207 | RR207 | PP-13 |
| 28 | RR28 | CMH-28 | 88 | RR88 | CMH-90 | 148 | RR148 | MLT-11 | 208 | RR208 | PP-14 |
| 29 | RR29 | CMH-31 | 89 | RR89 | CMH-91 | 149 | RR149 | MLT-12 | 209 | RR209 | PP-15 |
| 30 | RR30 | CMH-32 | 90 | RR90 | CMH-92 | 150 | RR150 | MLT-13 | 210 | RR210 | PP-17 |
| 31 | RR31 | CMH-33 | 91 | RR91 | CMH-93 | 151 | RR151 | MLT-14 | 211 | RR211 | PP-18 |
| 32 | RR32 | CMH-34 | 92 | RR92 | CMH-94 | 152 | RR152 | MLT-15 | 212 | RR212 | PP-19 |
| 33 | RR33 | CMH-35 | 93 | RR93 | CMH-95 | 153 | RR153 | MLT-16 | 213 | RR213 | PP-20 |
| 34 | RR34 | CMH-36 | 94 | RR94 | CMH-96 | 154 | RR154 | MLT-17 | 214 | RR214 | PP-21 |
| 35 | RR35 | CMH-37 | 95 | RR95 | CMH-97 | 155 | RR155 | MLT-18 | 215 | RR215 | PP-22 |
| 36 | RR36 | CMH-38 | 96 | RR96 | CMH-98 | 156 | RR156 | MLT-19 | 216 | RR216 | PP-23 |
| 37 | RR37 | CMH-39 | 97 | RR97 | CMH-99 | 157 | RR157 | MLT-20 | 217 | RR217 | PP-24 |
| 38 | RR38 | CMH-40 | 98 | RR98 | CMH-100 | 158 | RR158 | MLT-21 | 218 | RR218 | PP-25 |
| 39 | RR39 | CMH-41 | 99 | RR99 | CMH-101 | 159 | RR159 | MLT-22 | 219 | RR219 | PP-27 |
| 40 | RR40 | CMH-42 | 100 | RR100 | CMH-103 | 160 | RR160 | MLT-23 | 220 | RR220 | PP-28 |
| 41 | RR41 | CMH-43 | 101 | RR101 | CMH-104 | 161 | RR161 | MLT-24 | 221 | RR221 | PP-29 |
| 42 | RR42 | CMH-44 | 102 | RR102 | CMH-105 | 162 | RR162 | MLT-25 | 222 | RR222 | PP-31 |
| 43 | RR43 | CMH-45 | 103 | RR103 | CMH-106 | 163 | RR163 | MLT-26 | 223 | RR223 | PP-32 |
| 44 | RR44 | CMH-46 | 104 | RR104 | CMH-107 | 164 | RR164 | MLT-27 | 224 | RR224 | PP-33 |
| 45 | RR45 | CMH-47 | 105 | RR105 | CMH-108 | 165 | RR165 | MLT-28 | 225 | RR225 | PP-34 |
| 46 | RR46 | CMH-48 | 106 | RR106 | CMH-109 | 166 | RR166 | MLT-29 | 226 | RR226 | PP-35 |
| 47 | RR47 | CMH-49 | 107 | RR107 | CMH-110 | 167 | RR167 | MLT-30 | 227 | RR227 | PP-36 |
| 48 | RR48 | CMH-50 | 108 | RR108 | CMH-111 | 168 | RR168 | MLT-31 | 228 | RR228 | PP-37 |
| 49 | RR49 | CMH-51 | 109 | RR109 | CMH-112 | 169 | RR169 | MLT-32 | 229 | RR229 | PP-38 |
| 50 | RR50 | CMH-52 | 110 | RR110 | CMH-113 | 170 | RR170 | MLT-33 | 230 | RR230 | PP-39 |
| 51 | RR51 | CMH-53 | 111 | RR111 | CMH-114 | 171 | RR171 | MLT-34 | 231 | RR231 | PP-40 |
| 52 | RR52 | CMH-54 | 112 | RR112 | CMH-115 | 172 | RR172 | MLT-35 | 232 | RR232 | PP-41 |
| 53 | RR53 | CMH-55 | 113 | RR113 | CMH-116 | 173 | RR173 | MLT-36 | 233 | RR233 | PP-42 |
| 54 | RR54 | CMH-56 | 114 | RR114 | CMH-117 | 174 | RR174 | MLT-37 | 234 | RR234 | PP-43 |
| 55 | RR55 | CMH-57 | 115 | RR115 | CMH-118 | 175 | RR175 | MLT-38 | 235 | RR235 | PP-45 |
| 56 | RR56 | CMH-58 | 116 | RR116 | CMH-119 | 176 | RR176 | MLT-39 | 236 | RR236 | PP-46 |
| 57 | RR57 | CMH-59 | 117 | RR117 | CMH-120 | 177 | RR177 | MLT-40 | 237 | RR237 | PP-47 |
| 58 | RR58 | CMH-60 | 118 | RR118 | CMH-121 | 178 | RR178 | MLT-41 | 238 | RR238 | PP-48 |
| 59 | RR59 | CMH-61 | 119 | RR119 | CMH-122 | 179 | RR179 | MLT-42 | 239 | RR239 | PP-51 |
| 60 | RR60 | CMH-62 | 120 | RR120 | CMH-123 | 180 | RR180 | MLT-43 | 240 | RR240 | PP-53 |

Table S2. Details of the molecular markers used for screening of restorer lines for restorer gene identification

| Marker | Rf gene | Marker type | Primer sequence | Expected Allele size (bp) | Reference |
| --- | --- | --- | --- | --- | --- |
| DRRM‐Rf3‐5 | *Rf3* | Gene‐based | F: GATGGCACAGCTTCAGAACA  R: CTAATTCTGGGCGAGCAAAG | 160 (F)  130 (M) | Suresh et al., 2012 |
| RM 6100 | *Rf4* | Gene‐linked | F: TCCTCTACCAGTACCGCACC  R: GCTGGATCACAGATCATTGC | 150 (F)  140 (M) | Prakash et al., 2003 |

Table S3. Molecular characterization of 240 rice restorer lines for fertility restorer genes (*Rf3* and *Rf4*)

| **Genotype** | ***Rf3*** | ***Rf4*** | **Genotype** | ***Rf3*** | ***Rf4*** | **Genotype** | ***Rf3*** | ***Rf4*** | **Genotype** | ***Rf3*** | ***Rf4*** |
| --- | --- | --- | --- | --- | --- | --- | --- | --- | --- | --- | --- |
| RR1 | N | P | RR61 | N | P | RR121 | N | P | RR181 | N | P |
| RR2 | N | P | RR62 | N | P | RR122 | N | P | RR182 | N | P |
| RR3 | P | P | RR63 | N | P | RR123 | N | P | RR183 | N | P |
| RR4 | P | P | RR64 | N | P | RR124 | N | P | RR184 | N | P |
| RR5 | N | P | RR65 | N | P | RR125 | N | P | RR185 | N | P |
| RR6 | N | P | RR66 | N | P | RR126 | N | P | RR186 | N | R |
| RR7 | N | P | RR67 | N | P | RR127 | N | P | RR187 | N | P |
| RR8 | N | P | RR68 | N | P | RR128 | N | P | RR188 | N | P |
| RR9 | N | P | RR69 | P | P | RR129 | N | P | RR189 | N | R |
| RR10 | N | P | RR70 | N | P | RR130 | N | P | RR190 | N | P |
| RR11 | N | P | RR71 | N | P | RR131 | N | P | RR191 | N | P |
| RR12 | N | P | RR72 | N | P | RR132 | N | P | RR192 | N | P |
| RR13 | P | P | RR73 | N | P | RR133 | N | P | RR193 | N | P |
| RR14 | N | P | RR74 | N | P | RR134 | N | P | RR194 | N | P |
| RR15 | N | P | RR75 | N | H | RR135 | N | P | RR195 | N | P |
| RR16 | N | P | RR76 | N | P | RR136 | N | P | RR196 | N | P |
| RR17 | N | P | RR77 | N | P | RR137 | N | P | RR197 | N | P |
| RR18 | N | P | RR78 | N | P | RR138 | N | P | RR198 | N | P |
| RR19 | N | P | RR79 | N | P | RR139 | N | P | RR199 | N | P |
| RR20 | N | P | RR80 | N | P | RR140 | N | P | RR200 | N | P |
| RR21 | N | P | RR81 | N | P | RR141 | N | P | RR201 | N | P |
| RR22 | N | P | RR82 | N | P | RR142 | N | P | RR202 | P | P |
| RR23 | N | P | RR83 | N | P | RR143 | N | P | RR203 | N | P |
| RR24 | N | P | RR84 | N | P | RR144 | N | P | RR204 | P | P |
| RR25 | N | P | RR85 | N | P | RR145 | N | P | RR205 | N | P |
| RR26 | N | P | RR86 | N | P | RR146 | N | P | RR206 | P | N |
| RR27 | N | P | RR87 | P | P | RR147 | N | P | RR207 | P | P |
| RR28 | N | P | RR88 | N | P | RR148 | N | H | RR208 | N | P |
| RR29 | N | P | RR89 | N | P | RR149 | R | N | RR209 | N | P |
| RR30 | N | P | RR90 | N | P | RR150 | N | P | RR210 | N | P |
| RR31 | N | P | RR91 | N | P | RR151 | N | P | RR211 | P | P |
| RR32 | N | P | RR92 | N | P | RR152 | P | P | RR212 | N | P |
| RR33 | N | P | RR93 | N | P | RR153 | N | P | RR213 | N | P |
| RR34 | N | P | RR94 | P | H | RR154 | N | P | RR214 | P | P |
| RR35 | N | P | RR95 | N | P | RR155 | N | P | RR215 | N | P |
| RR36 | P | P | RR96 | N | P | RR156 | N | P | RR216 | P | P |
| RR37 | N | P | RR97 | N | P | RR157 | N | P | RR217 | P | P |
| RR38 | N | P | RR98 | N | P | RR158 | P | P | RR218 | P | P |
| RR39 | N | H | RR99 | N | P | RR159 | N | P | RR219 | P | P |
| RR40 | N | P | RR100 | N | P | RR160 | N | P | RR220 | P | P |
| RR41 | N | P | RR101 | N | P | RR161 | N | P | RR221 | N | P |
| RR42 | N | H | RR102 | N | P | RR162 | N | P | RR222 | N | P |
| RR43 | N | H | RR103 | N | P | RR163 | N | P | RR223 | N | P |
| RR44 | N | P | RR104 | N | P | RR164 | N | P | RR224 | P | P |
| RR45 | N | P | RR105 | N | P | RR165 | N | H | RR225 | P | P |
| RR46 | N | P | RR106 | P | P | RR166 | N | P | RR226 | P | P |
| RR47 | N | P | RR107 | P | P | RR167 | N | P | RR227 | N | P |
| RR48 | N | P | RR108 | N | P | RR168 | N | P | RR228 | N | P |
| RR49 | N | P | RR109 | N | P | RR169 | N | P | RR229 | N | P |
| RR50 | N | P | RR110 | N | P | RR170 | N | P | RR230 | N | P |
| RR51 | N | P | RR111 | N | P | RR171 | N | P | RR231 | N | P |
| RR52 | N | P | RR112 | N | P | RR172 | N | P | RR232 | P | N |
| RR53 | N | P | RR113 | N | P | RR173 | N | P | RR233 | P | P |
| RR54 | N | P | RR114 | N | P | RR174 | N | P | RR234 | P | N |
| RR55 | N | P | RR115 | N | P | RR175 | N | P | RR235 | P | P |
| RR56 | N | P | RR116 | N | P | RR176 | N | P | RR236 | P | P |
| RR57 | N | P | RR117 | N | P | RR177 | N | P | RR237 | P | N |
| RR58 | N | P | RR118 | N | P | RR178 | N | P | RR238 | P | N |
| RR59 | N | P | RR119 | N | P | RR179 | N | P | RR239 | P | N |
| RR60 | N | P | RR120 | N | P | RR180 | N | P | RR240 | P | P |

*Rf_3_* and *Rf_4_* allele status was scored based on the marker profiles. P = presence of the fertility restoration allele; N = absence of the fertility restoration allele (maintainer-type allele); H = heterozygous state (presence of both restoration and non-restoration alleles). All genotypes were classified consistently using this coding scheme across the supplementary tables.

Table S4 Performance and stability metrics of top-ranking restorer lines identified through WAASBY analysis

| SN | Genotypes (a) | WAASBY index | Genotypes (b) | WAASB Index |
| --- | --- | --- | --- | --- |
| 1 | RR196 | 77.9 | RR196 | 0.053 |
| 2 | RR197 | 73.5 | RR197 | 0.221 |
| 3 | RR101 | 73.3 | RR101 | 0.168 |
| 4 | RR137 | 71.5 | RR137 | 0.202 |
| 5 | RR100 | 71.1 | RR100 | 0.0292 |
| 6 | RR23 | 70.7 | RR23 | 0.0608 |
| 7 | RR146 | 70.4 | RR146 | 0.119 |
| 8 | RR140 | 70.3 | RR201 | 0.028 |
| 9 | RR201 | 70.1 | RR74 | 0.0686 |
| 10 | RR74 | 70 | RR17 | 0.122 |
| 11 | RR17 | 69.9 | RR42 | 0.0585 |
| 12 | RR42 | 69.6 | RR151 | 0.147 |
| 13 | RR151 | 69.6 | RR15 | 0.0436 |
| 14 | RR15 | 69.4 | RR199 | 0.169 |
| 15 | RR199 | 69.3 | RR14 | 0.31 |
| 16 | RR14 | 69.2 | RR231 | 0.144 |
| 17 | RR231 | 69.2 | RR19 | 0.266 |
| 18 | RR19 | 69.2 | RR126 | 0.301 |
| 19 | RR126 | 69.1 | RR195 | 0.157 |
| 20 | RR195 | 69 | RR185 | 0.128 |
| 21 | RR185 | 68.9 | RR105 | 0.0848 |
| 22 | RR105 | 68.9 | RR104 | 0.0338 |
| 23 | RR104 | 68.8 | RR67 | 0.131 |
| 24 | RR67 | 68.8 |  |  |

a: Ranking of top 10% of the restorers based on WAASBY values; b: Top restorers in Quadrant IV of Y x WAASB biplot; WAASB: Weighted Average of Absolute Scores from the singular value decomposition (SVD) of the matrix of Best Linear Unbiased Prediction; WAASBY: WAASB x Mean Yield

Table S5 List of selected rice restorers in different multi-trait selection indices

| **SN** | **MGIDI** | **MTSI** | **MTMPS** | **FAI_BLUP** |
| --- | --- | --- | --- | --- |
| 1 | RR130 | RR233 | RR140 | RR125 |
| 2 | RR226 | RR130 | RR196 | RR130 |
| 3 | RR125 | RR196 | RR58 | RR86 |
| 4 | RR133 | RR31 | RR79 | RR121 |
| 5 | RR197 | RR17 | RR73 | RR122 |
| 6 | RR131 | RR16 | RR111 | RR226 |
| 7 | RR72 | RR73 | RR130 | RR102 |
| 8 | RR140 | RR122 | RR195 | RR72 |
| 9 | RR120 | RR88 | RR198 | RR197 |
| 10 | RR121 | RR14 | RR23 | RR120 |
| 11 | RR30 | RR126 | RR134 | RR140 |
| 12 | RR137 | RR114 | RR125 | RR25 |
| 13 | RR102 | RR200 | RR14 | RR133 |
| 14 | RR79 | RR79 | RR61 | RR115 |
| 15 | RR86 | RR217 | RR74 | RR195 |
| 16 | RR122 | RR30 | RR17 | RR134 |
| 17 | RR115 | RR120 | RR112 | RR137 |
| 18 | RR134 | RR102 | RR122 | RR53 |
| 19 | RR196 | RR121 | RR121 | RR196 |
| 20 | RR240 | RR197 | RR102 | RR112 |
| 21 | RR58 | RR15 | RR72 | RR79 |
| 22 | RR195 | RR211 | RR31 | RR131 |
| 23 | RR116 | RR125 | RR12 | RR58 |
| 24 | RR200 | RR29 | RR119 | RR240 |
| 25 | RR153 | RR93 | RR114 | RR49 |
| 26 | RR25 | RR61 | RR16 | RR77 |
| 27 | RR144 | RR112 | RR236 | RR97 |
| 28 | RR127 | RR214 | RR233 | RR62 |
| 29 | RR112 | RR137 | RR50 | RR30 |
| 30 | RR77 | RR58 | RR105 | RR124 |
| 31 | RR124 | RR20 | RR36 | RR126 |
| 32 | RR128 | RR203 | RR203 | RR103 |
| 33 | RR233 | RR85 | RR120 | RR233 |
| 34 | RR126 | RR226 | RR35 | RR211 |
| 35 | RR9 | RR132 | RR32 | RR23 |
| 36 | RR71 | RR202 | RR20 | RR127 |

Table S6 Trait performance of superior genotypes identified from the study with stability for important traits using multi-trait stability models

| **Genotypes** | **DFF** | **PH** | **PER** | **SF** | **PL** | **PTN** | **TSW** | **FG** | **SYP** |
| --- | --- | --- | --- | --- | --- | --- | --- | --- | --- |
| RR23 | 107.83 | 111.37 | 115.94 | 90.45 | 26.47 | 11 | 21.75 | 143 | 23.11 |
| RR72 | 104.50 | 113.07 | 109.09 | 85.93 | 26.32 | 13 | 20.28 | 189 | 28.66 |
| RR79 | 95.50 | 108.53 | 108.83 | 88.49 | 26.38 | 11 | 22.55 | 162 | 21.01 |
| RR121 | 102.50 | 114.70 | 111.48 | 87.18 | 25.49 | 16 | 23.39 | 155 | 37.43 |
| RR130 | 104.17 | 111.10 | 104.36 | 88.33 | 25.82 | 12 | 24.36 | 151 | 29.97 |
| RR140 | 104.17 | 108.22 | 124.26 | 90.42 | 25.47 | 14 | 22.86 | 153 | 31.81 |
| RR196 | 102.17 | 109.57 | 111.26 | 86.44 | 25.39 | 11 | 24.28 | 157 | 26.62 |
| RR233 | 103.17 | 100.33 | 104.94 | 85.60 | 25.69 | 11 | 23.35 | 189 | 22.50 |

DFF = Days to 50% flowering (days), PH = Plant height in cm, PER=Panicle exertion ratio, PL = Panicle length in cm, SF= Spikelet fertility in %, PTN = Productive tillers number, FG = Filled grains per panicle, TSW = Thousand seed weight in g, SYP = Seed yield per plant in g.

Table S7 Monthly weather conditions during the conduct of experiment at the experimental locations

|  | **Delhi 2022** | | | **Rakhra 2022** | | | **Aduthurai 2023** | | |
| --- | --- | --- | --- | --- | --- | --- | --- | --- | --- |
| **Month** | **Mean maximum temperature (°C)** | **Mean minimum temperature (°C)** | **Total rainfall (mm)** | **Mean maximum temperature (°C)** | **Mean minimum temperature (°C)** | **Total rainfall (mm)** | **Mean maximum temperature (°C)** | **Mean minimum temperature (°C)** | **Total rainfall (mm)** |
| January | 17.37 | 7.49 | 141.90 | January | 16.82 | 8.91 | 29.8 | 19.90 | 6.80 |
| February | 23.14 | 8.74 | 30.00 | February | 22.08 | 8.79 | 31.41 | 19.54 | 70.40 |
| March | 32.31 | 15.30 | 0.00 | March | 31.77 | 16.55 | 34.06 | 22.44 | 25.80 |
| April | 40.06 | 19.81 | 0.00 | April | 40.11 | 21.24 | 36.05 | 24.51 | 0.00 |
| May | 39.77 | 24.80 | 60.00 | May | 38.76 | 25.28 | 35.85 | 25.92 | 58.50 |
| June | 40.44 | 26.03 | 88.80 | June | 39.69 | 26.51 | 36.37 | 25.59 | 79.8 |
| July | 35.07 | 26.09 | 325.40 | July | 34.18 | 26.92 | 35.66 | 25.61 | 14.8 |
| August | 34.14 | 25.55 | 79.30 | August | 34.6 | 27.10 | 36.49 | 26.44 | 120.9 |
| September | 34.16 | 24.05 | 191.40 | September | 33.65 | 25.09 | 34.77 | 24.61 | 31.4 |
| October | 31.30 | 19.97 | 134.90 | October | 32.09 | 18.81 | 34.14 | 24.65 | 17.2 |
| November | 28.05 | 14.37 | 0.00 | November | 28.18 | 12.61 | 30.70 | 23.88 | 270.6 |
| December | 22.61 | 6.69 | 0.00 | December | 21.4 | 8.12 | 29.90 | 22.68 | 79.2 |
